# Preclinical Evaluation of ON203, A Novel Bioengineered mAb Targeting Oxidized Macrophage Migration Inhibitory Factor as an Anticancer Therapeutic

**DOI:** 10.1158/1535-7163.MCT-22-0676

**Published:** 2023-04-17

**Authors:** Gregor Rossmueller, Irina Mirkina, Barbara Maurer, Verena Hoeld, Julia Mayer, Michael Thiele, Randolf J. Kerschbaumer, Alexander Schinagl

**Affiliations:** 1 OncoOne Research & Development GmbH, Vienna, Austria.

## Abstract

High levels of macrophage migration inhibitory factor (MIF) in patients with cancer are associated with poor prognosis. Its redox-dependent conformational isoform, termed oxidized MIF (oxMIF), is a promising tumor target due to its selective occurrence in tumor lesions and at inflammatory sites. A first-generation anti-oxMIF mAb, imalumab, was investigated in clinical trials in patients with advanced solid tumors, where it was well tolerated and showed signs of efficacy. However, imalumab has a short half-life in humans, increased aggregation propensity, and an unfavorable pharmacokinetic profile. Here, we aimed to optimize imalumab by improving its physicochemical characteristics and boosting its effector functions. Point mutations introduced into the variable regions reduced hydrophobicity and the antibodies’ aggregation potential, and increased plasma half-life and tumor accumulation *in vivo*, while retaining affinity and specificity to oxMIF. The introduction of mutations into the Fc region known to increase antibody-dependent cellular cytotoxicity resulted in enhanced effector functions of the novel antibodies *in vitro*, whereas reduced cytokine release from human peripheral blood mononuclear cells in the absence of target antigen by the engineered anti-oxMIF mAb ON203 versus imalumab reveals a favorable *in vitro* safety profile. *In vivo*, ON203 mAb demonstrated superior efficacy over imalumab in both prophylactic and established prostate cancer (PC3) mouse xenograft models. In summary, our data highlight the potential of the second-generation anti-oxMIF mAb ON203 as a promising immunotherapy for patients with solid tumors, warranting clinical evaluation.

## Graphical Abstract

**Figure:**
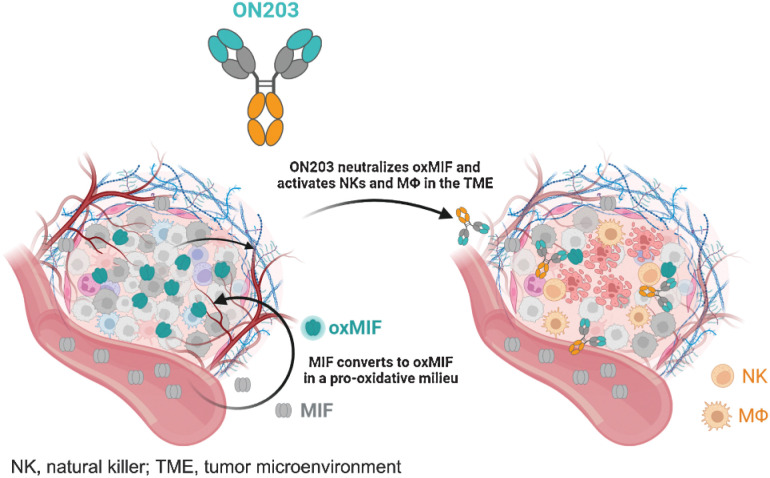

## Introduction

Macrophage migration inhibitory factor (MIF) is an evolutionarily conserved protein that shows a remarkable functional diversity, including specific binding to surface CD74 and chemokine receptors, presence of intrinsic tautomerase and oxidoreductase activities (1–3), and proinflammatory, antiapoptotic, proangiogenic, and proproliferative functions (4–6). Moreover, MIF is often overexpressed in tumor tissue from numerous cancer indications, which has been associated with higher tumor burden and grade, increased metastasis risk, and poor prognosis (7–9). Because of its ubiquitous nature, constitutive expression, and high occurrence in circulation of healthy subjects at levels of approximately 6 ng/mL (10), MIF can be regarded as an inappropriate target for drug development. Indeed, no small molecules, nor biologics selectively targeting MIF—including anti-MIF antibodies—have been approved.

However, MIF occurs in two immunologically distinct conformational isoforms, reduced MIF (redMIF) and oxidized MIF (oxMIF; ref. 10). Reduced MIF is the abundantly expressed isoform of MIF (10–12). In contrast, oxMIF is the disease-related isoform that was detected in patients with acute and chronic inflammatory diseases and is specifically present in solid tumors (10, 11), which renders oxMIF an attractive target for therapeutic intervention (13). Only recently, several groups confirmed that MIF is subject to posttranslational modification, particularly redox-dependent modification of the catalytic proline 1 and cysteine 80, regulating biological activity of MIF (12, 14). Skeens and colleagues used nuclear magnetic resonance (NMR) and mass spectrometry to substantiate earlier findings that in an oxidative environment MIF is structurally modified and becomes increasingly dynamic; MIF conformation and functional response may be altered in a redox-dependent manner (15).

The first-generation human IgG1 anti-oxMIF mAb (imalumab) was identified from a large and highly diverse panel of 145 unique MIF-specific antibodies that were selected from Dyax Fab310 phage display human Fab library (16). Of these 145 mAbs, only a few—BaxB01, BaxD08, BaxG03, and the affinity matured version of the latter, BaxM159—exerted protective effects in *in vivo* experimental sepsis, contact hypersensitivity, colitis, prostate cancer and ovarian cancer xenograft mouse models, and glomerulonephritis rat models (10, 11, 16, 17). These *in vivo* effective mAbs bind to two regions of MIF, amino acids 50–68 [epitope of BaxB01 (RCSB PDB: 6FOE) sharing the same variable region with Bax69 = imalumab] or amino acids 86–102 (epitope of BaxG03 and BaxM159), that form a *β*-barrel structure harboring the MIF oxidoreductase motif (16) and these antibodies selectively bind oxMIF, but not redMIF (10).

Imalumab (Bax69) was investigated in a phase I study (NCT01765790) in patients with advanced solid tumors (schedule 1) and in patients with metastatic colorectal carcinoma (mCRC), non–small cell lung cancer, and ovarian cancer (schedule 2), where it demonstrated an acceptable safety profile and exerted some antitumor activity (stable disease in 26% of the patient population; ref. 18). In addition, imalumab was assessed in a phase I/IIa monotherapy study in patients with malignant ascites of ovarian cancer (NCT02540356) and in a phase IIa combination study in patients with mCRC (NCT02448810). However, both studies were terminated prematurely based on inadequate design and an overall benefit-risk assessment, respectively (18). Imalumab exhibits an unusually short half-life of only 56–176 hours in humans and showed a nonlinear pharmacokinetic dose relationship, indicating some intrinsic limitations of this first-generation anti-oxMIF antibody (18, 19). Furthermore, first-generation anti-oxMIF mAbs did not show any antibody-dependent cellular cytotoxicity (ADCC) activity *in vitro* (17).

Several *in vitro* and *in vivo* studies have demonstrated a crucial role of ADCC and antibody-dependent cellular phagocytosis (ADCP) in tumor elimination by immune cells (20–26). The antibody Fc region, specifically the CH2 domain, can be mutated to increase Fc affinity to the activatory human Fcγ receptors (FcγR), particularly FcγRIIIA and FcγRIIA (23, 26). Within the IgG subclass, human IgG1 is the most effective at inducing ADCC by natural killer (NK) cells (27). Panitumumab (anti-EGFR, IgG2) did not elicit any ADCC by NK cells, but it potently induced ADCC by myeloid cells (monocytes/macrophages and neutrophils) via their FcγRIIA (27, 28). Several CH2 mutations combined with isotypic modifications (IgG1/2 hybrid) were shown to be particularly efficient in inducing ADCC (29, 30). Human IgG1 binds with greater affinity to the V158/V158 allotype of FcγRIIIA than to F158/F158 (21, 25, 31). This difference in affinity and subsequent ADCC are significant determinants for efficacy of rituximab (anti-CD20, IgG1) therapy (21). Two recently approved Fc-engineered mAbs, margetuximab (anti-Her2) and tafasitamab (anti-CD19), are examples of successful ADCC enhancement against both allotypes of FcγRIIIA (22, 32).

Here, we describe the engineering and evaluation of second-generation anti-oxMIF mAbs with highly improved physicochemical and biological properties compared with imalumab. It is well known that controlling hydrophobicity and aggregation can improve antibody stability, manufacturability, formulation, and safety (33, 34). We identified residues in the variable domains of imalumab that contribute to a high surface hydrophobicity and aggregation propensity, which potentially led to the short half-life and limited efficacy. By introducing well-selected point mutations in the variable regions, we reduced hydrophobicity and aggregation propensity and improved the pharmacokinetic profile (i.e., extend plasma half-life and tumor accumulation) of the second-generation anti-oxMIF antibodies. Because imalumab, a fully human IgG1, was reported to be devoid of effector functions (17), we aimed to introduce potent effector functions via mutations in the heavy-chain constant domain of imalumab. Novel variable domains combined with an IgG1/IgG2 hybrid heavy-chain constant region carrying two point mutations (S239D/I332E) in the CH2 domain were intended to improve the manufacturability of the antibodies as well as enhance ADCC activity. The beneficial effects of the bioengineering efforts were confirmed *in vitro* by Peripheral blood mononuclear cell (PBMC)-mediated ADCC assays and *in vivo* by enhanced tumor growth inhibition in both prophylactic and therapeutic settings in prostate cancer (PC3) mouse xenograft models.

## Materials and Methods

### Expression and purification of human and mouse MIF

SHuffle T7 Express lysY Competent *E. coli* (New England BioLabs) were transfected with a Champion pET303/CT-His plasmid (Thermo Fisher Scientific, catalog no. K630203) containing either human or mouse MIF cDNA. The cDNA sequences were taken from uniprot.org under accession number P14174 and P34884 for human and mouse MIF, respectively. MIF is expressed by an inducible T7 promoter under the control of a lac operator. The plasmid possesses the bacterial antibiotic resistance gene AmpR under the weak AmpR promoter for clone selection with ampicillin. LB low salt broth (LLG Labware) made according to the manufacturer's instructions including 100 μg/mL ampicillin (Sigma-Aldrich) was used for the start of the expression. The culture was incubated at 37°C overnight shaking at 180 rpm. Super Optimal Broth (SOB) broth (Amresco) was made according to the manufacturer's instructions with the addition of 10 mmol/L MgCl_2_ (Sigma-Aldrich), 10 mmol/L MgSO_4_ (Honeywell), 2.5 mmol/L KCl (Chem-Lab), and 100 μg/mL ampicillin. The SOB was sterile filtered and inoculated with 1/100 of the volume of the overnight LB broth culture. After the media change, the cell suspension was incubated for around 24 hours at 30°C while shaking at 180 rpm. On the following day, the cells were harvested by centrifugation at 18,000 × *g* for 10 minutes at 4°C. The pellet was dissolved in 5 mL B-PER lysis solution (Thermo Fisher Scientific) containing 1 mmol/L ethylenediaminetetraacetic acid (EDTA; PanReac AppliChem) per g of cell pellet. The suspension was incubated for 15 minutes at room temperature and subsequently centrifuged for 20 minutes at 18,000 × *g* at 4°C. The supernatant contains MIF and was collected. An anion exchange chromatography at pH 8.0 using HiTrap DEAE FF columns (Cytiva) was used for the removal of host cell proteins. The sample was diluted with 1/10 volume of 10X DEAE loading buffer (200 mmol/L TRIS, pH 8.0). The columns were equilibrated with 3 column volumes (CV) of DEAE loading buffer and after application of the sample the bound proteins were eluted with DEAE elution buffer (20 mmol/L TRIS, 1 mol/L NaCl, pH 8.0). The flow-through (FT) contained MIF and was collected. In the next step, MIF was captured by cation exchange chromatography at pH 6.0 using HiTrap SP FF columns (Cytiva). The final FT from the DEAE purification was pH adjusted by adding 10X SP loading buffer (200 mmol/L Bis-TRIS, pH 6.3) and finally titrated to pH 6.3 with 0.1 mol/L HCl. The columns were equilibrated with 3 CV of SP loading buffer. The sample was applied with a residence time of 7.5 minutes. After washing the column with SP loading buffer, the MIF containing fraction was eluted with 10% SP elution buffer (20 mmol/L Bis-TRIS, 1 mol/L NaCl, pH 6.3). The column was regenerated by eluting the contaminations with 100% SP elution buffer. The MIF fraction was buffer exchanged to 1× Dulbecco's PBS (DPBS; Gibco) either using size exclusion chromatography (SEC) on a Superdex Increase 75 10/300 GL (Cytiva) column or Bio-Scale P6 desalting column (Cytiva).

### Reagents

TNB-MIF was produced as described previously (12) and polyclonal anti-MIF antibodies were generated by immunization of rabbits with human MIF in Complete Freund's Adjuvant, and anti-MIF antibodies were purified from serum by MabSelect PrismA columns (protein A) and HiTrap NHS activated columns (Cytiva) functionalized with recombinant human MIF according to the manufacturer's protocol.

### *De novo* gene synthesis and cloning of mAbs

The cDNAs of the mAb full-length heavy and light chains (Supplementary Table S1) were *de novo* gene synthesized at GeneArt/Thermo Fisher Scientific (with sequence codon optimization for *Cricetulus griseus*) and subcloned into the pcDNA 3.4 TOPO mammalian expression vector (Invitrogen, catalog no. A14697) in-frame with the N-terminal signal peptide sequence METDTLLLWVLLLWVPGSTG.

### mAb expression and purification

The antibodies were expressed using the ExpiCHO Expression system (Thermo Fisher Scientific, catalog no. A29133, RRID:CVCL 5J31) for transient protein expression. The manufacturer's expression max titer protocol was strictly followed. The supernatants were harvested after 13–14 days of production by centrifugation and the mAbs were purified by Protein A chromatography using MabSelect Prism A columns (Cytiva) with 25 mmol/L Tris, 25 mmol/L NaCl buffer (pH 7.1) for equilibration and 100 mmol/L glycine, 50 mmol/L NaCl buffer (pH 3.5) for elution. The eluates were immediately neutralized by adding 400 mmol/L glycine buffer (pH 8.1) or 500 mmol/L 2-(N-morpholino) ethanesulfonic acid (MES), pH 5.8. As a polishing step, antibodies (C0008, which is a mAb produced in our lab with the identical sequence as imalumab but devoid of the C-terminal lysine, ON103, or ON203; Supplementary Table S1) were loaded onto HiTrap SP HP columns (Cytiva) and eluted stepwise by 50 mmol/L MES buffer (pH 6.0) containing NaCl. The antibodies were buffer exchanged to a storage buffer and sterile filtered before storing them at −20°C until further use.

### Anti-oxMIF mAb ELISA

The anti-oxMIF mAb ELISA was performed as described previously (35) with the following modifications: 100 μL/well recombinant MIF at 1 μg/mL diluted in DPBS (Thermo Fisher Scientific) was immobilized on Nunc MaxiSorp 96-well plates (Thermo Fisher Scientific) for 1 hour at room temperature (immobilization leads to a conformational change in MIF structure to expose anti-oxMIF mAb epitopes; ref. 10). Wells were blocked with 250 μL 2% fish gelatine (w/v; Sigma-Aldrich) in TBST [50 mmol/L Tris, 150 mmol/L NaCl, 0.1% Tween20 (v/v), pH 7.5] (referred to as dilution buffer) overnight at 4°C. Serial dilutions for antibodies in dilution buffer were added to the plates and incubated at room temperature for 1 hour. The bound antibodies were detected by incubation with 100 μL/well of a goat anti-human IgG Fc cross-adsorbed horseradish peroxidase (HRP) conjugate at 25 ng/mL (Thermo Fisher Scientific, # A18823, RRID:AB_2535600) for 1 hour at room temperature and tetramethylbenzidine (Thermo Fisher Scientific) as substrate. The reaction was stopped with 50 μL/well 30% sulfuric acid (v/v) and the absorbance was measured at 450 and 650 nm using a Tecan Infinite M200 PRO microplate reader (RRID:SCR_019033). For data analysis, the absorbance values at 650 nm were subtracted from the values at 450 nm. Data from different experiments were normalized to the maximal absorbance of C0008 (=100%) of the respective experiment and EC_50_ values, as estimates for apparent affinity, were calculated by nonlinear regression variable slope four-parameter logistic curve (4PL) fitting using the following equation in GraphPad Prism (RRID:SCR_002798): Y = Bottom + (Top-Bottom)/(1+10^((logEC_50_-X)*HillSlope)), where X is the concentration of agonist (antibody), Y is the response, and HillSlope is a slope factor.

### Differential oxMIF-binding ELISA

Determination of oxMIF specificity was done according to Schinagl and colleagues (12) with the following modifications. Antibodies were immobilized at 15 nmol/L in 100 μL/well and 50 ng/mL TNB-MIF was used as an oxMIF surrogate.

### Quantitative oxMIF ELISA

Determination of anti-oxMIF antibody affinity was done according to Thiele and colleagues (10) with the following modifications. Fifteen nmol/L C0008, ON103 and ON203 (produced at OncoOne) were used as capture antibodies instead of BaxM159 (10). Human or mouse MIF in 0.2% Proclin300 containing buffers were used to create standard curves and EC_50_ values were calculated as described earlier.

### Affinity determination by surface plasmon resonance using immobilized anti-oxMIF mAbs

The second-generation anti-oxMIF antibodies ON103 and ON203, as well as the reference anti-oxMIF mAb C0008 were immobilized to Biacore CM5 optical sensor chips (Cytiva, #29149604) using standard amine coupling conditions (Cytiva, Amine Coupling Kit, #BR100050) to a density of approximately 2,500; 1,500; or 1,000 RU on flow cells FC2, FC3, and FC4, respectively. FC1 was used as a reference cell and was only reacted with buffers. Mouse or human recombinant MIF were diluted in HBS-EP buffer (Cytiva, # BR100188) to concentrations of 42–666 nmol/L in the presence of 0.2% Proclin300 (Sigma-Aldrich, 48912-U), to transform MIF into an oxMIF surrogate (10) for 3 hours at room temperature. Proclin300-treated MIF was applied to immobilized anti-oxMIF antibodies at a flow rate of 30 μL/minute for 3 minutes using a Biacore 3000 Instrument (Cytiva, RRID:SCR_018044). The dissociation time was 300 seconds, and the chip was regenerated using 10 mmol/L HCl for 3 minutes. The kinetics of the concentration series were analyzed by local simultaneous association/dissociation fitting of each binding curve after subtraction of background from the reference cell (FC1) using the iterative Langmuir 1:1 interaction model with mass transfer compensation provided by the BiaEvaluation 4.1 software (Cytiva, RRID:SCR_015936). Data are represented as mean ± SD of the calculated association constant (ka), dissociation constant (kd), and equilibrium constant (K_D_) values of flow cells 2–4.

### Hydrophobic interaction chromatography

Antibodies were diluted to 1 mg/mL with 50 mmol/L phosphate, 750 mmol/L ammonium sulphate buffer, pH 6.9, and were loaded onto a 1 mL HiTrap Butyl HP column (Cytiva, # 28411001). The column was equilibrated with 50 mmol/L phosphate, 1.125 mol/L ammonium sulphate, pH 7.0, and the analysis was carried out over a 20-column volume linear gradient from 0% to 100% 50 mmol/L phosphate, 20% isopropanol, pH 7.0. All samples were analyzed by measuring absorbance at 280 nm on an NGC Quest 10 system (Bio-Rad) and retention volumes were determined by ChromLab Software (Bio-Rad, RRID:SCR_008426).

### Epitope mapping

The full linear sequence of human macrophage MIF (UniProt ID P14174) was converted into a library of 15mer peptides each overlapping by 1 amino acid and directly synthesized on an amino-functionalized solid support. Peptides were synthesized by Fmoc-based solid-phase peptide synthesis and the peptide arrays were incubated with 25 ng/mL of primary antibodies ON103, ON203 or an isotype IgG (1 μg/mL) overnight at 4°C. After washing, the peptide arrays were incubated with a 1/1,000 dilution of a goat anti-human HRP conjugate (Southern Biotech, # 2010-05, RRID:AB_2795564) for 1 hour at 25°C. After washing, the peroxidase substrate 2,2′-azino-di-3-ethylbenzthiazoline sulfonate and 20 μL/mL of 3% H_2_O_2_ were added. After 1 hour, the color was quantified with a charge-coupled device [milli absorption units (mAU) range, 0–3,000]—camera and image processing system. Data are presented as mAU per 15mer peptide.

### Cell lines

HCT116 (RRID:CVCL 0291) human colorectal carcinoma cell line established from an adult male was obtained from Sigma (ECACC, # 91091005) in 2019 or was obtained from NCI (DCTD Tumor Repository, NCI-60 Cancer Cell Line Panel) for animal experiments. PC-3 (RRID:CVCL_0035) human prostate adenocarcinoma established from an adult male was obtained from Sigma (ECACC, # 90112714) in 2019 or was obtained from NCI (DCTD Tumor Repository, NCI-60 Cancer Cell Line Panel) for animal experiments. CT26 (RRID:CVCL_7254) mouse carcinoma cell line established from a female Balb/c mouse was purchased from ATCC (# ATCC-CRL-2638). Cells were maintained at 37°C/5% CO2 in RPMI1640 medium (Gibco, # 42401042) supplemented with 10% heat-inactivated FBS (Gibco, # 16140071), 2 mmol/L L-Glutamine (Gibco, # 25030081), 100 U/mL penicillin, and 100 μg/mL streptomycin (Gibco, # 15140122). Subconfluent cultures (70%–80%) were split into new tissue culture flasks 1:2 to 1:10, that is, seeding at 1–5 × 10^4^ cells/cm^2^, using TrypLE Express Enzyme (Thermo Fisher Scientific, # 12605010) twice a week. Cells were routinely tested for *Mycoplasma* species by PCR at the respective cell bank collection (ATCC, ECACC, or NCI) or at Charles River Laboratories Germany GmbH (Freiburg) and were used in experiments between passage 6 and passage 10. Cell morphology was confirmed by microscopy.

### Expression of oxMIF at the cell surface of PC-3 and HCT116 cancer cell lines

Expression of oxMIF at the cell surface of human prostate adenocarcinoma cell line PC-3 (RRID:CVCL_0035, ECACC, Sigma catalog no. 90112714) and colon carcinoma cell line HCT116 (RRID:CVCL_0291, ECACC, Sigma catalog no. 91091005) was assessed by flow cytometry. Cells were dislodged from flasks by treatment with CellStripper nonenzymatic cell dissociation buffer (VWR, catalog no. 25-056-CI) and were stained with anti-oxMIF mAbs, or isotype IgG (palivizumab, Abbvie, catalog no. PZN-10974950) at 75 nmol/L in PBS supplemented with 5% BSA for 45 minutes at 4°C. After washing with PBS+5% BSA, cells were stained with a polyclonal goat anti-human IgG (H+L) Alexa Fluor488-conjugated antibody (Thermo Fisher Scientific, catalog no. A11013, RRID:AB_2534080) diluted 1:200 in PBS+5%BSA for 40 minutes at 4°C for the detection of oxMIF-bound mAbs. Cells were next washed with PBS and stained with eBioscience Fixable Viability Dye eFluor 780 (Thermo Fisher Scientific, # 65-0865-14) diluted in PBS (1:2,000) for 20 minutes at 4°C. After washing with PBS supplemented with 5% BSA, cells were fixed with eBioscience IC Fixation Buffer (Thermo Fisher Scientific, catalog no. 00-8222-49) for 10 minutes at 4°C. After washing, cells were measured on a CytoFlex-S flow cytometer (Beckman Coulter, RRID:SCR_019627, and data were analyzed by using FlowJo software (BD, RRID:SCR_008520).

### Cloning of human MIF into pDisplay vector and generation of HCT116/pMIF and HCT116/HiBiT/pMIF stable cell lines

The cDNA encoding human MIF (UniProtKB/Swiss-Prot accession number P14174, amino acid residues 2–115) was cloned into the pDisplay mammalian expression vector (Invitrogen/Thermo Fisher Scientific, # V66020) in-frame with N-terminal Hemagglutinin A epitope and C-terminal Myc-epitope by using B*gl* II (5′-) and *Pst* I (3′-) cloning sites. Because of the C-terminal transmembrane anchoring domain of platelet-derived growth factor receptor (PDGFR), after the removal of the signal peptide (IgG k-chain leader sequence), the monomeric huMIF-Myc-PDGFR fusion protein expressed from this construct is designed to be exposed at the cell surface of the transfected mammalian cells. HCT116 cells (RRID:CVCL_0291, ECACC-91091005) were transfected with the CAG/HaloTag-HiBiT/BlastR plasmid (Promega, # CS1956B17), selected with blasticidin and sorted as cell pools (HCT116/HiBiT) on MoFlow Astrios cell sorter (Beckman Coulter, RRID:SCR_019615). Both parental HCT116 cells and stable HiBiT-expressing cell line were then transfected with a huMIF-pDisplay plasmid, and stable clones carrying huMIF-pDisplay plasmid were selected with G418 and sorted using BD FACSAria IIIu (RRID:SCR_016695) sorter to generate cell lines stably expressing membrane-anchored monomeric MIF (termed HCT116/pMIF), or membrane-anchored monomeric MIF and intracellular HiBiT (termed HCT116/HiBiT/pMIF). Sorting was repeated to enrich for pMIF- and HiBiT/pMIF-positive clones. Expression of the cell surface huMIF (±intracellular HiBiT) in stable clones was assessed by flow cytometry. For this, HCT116/pMIF and HCT116/HiBiT/pMIF cells were stained with Janelia Fluor 646-conjugated HaloTag Ligand (Promega, # GA111A) according to the manufacturer's instructions to allow the visualization of HiBiT-tagged HaloTag protein. Subsequently, cells were stained with a polyclonal rabbit anti-MIF antibody (OncoOne) and a goat anti-rabbit (H+L) Alexa Fluor488-conjugated antibody (Thermo Fisher Scientific, # A11034, RRID:AB_2576217) for the detection of human MIF. Cells were acquired on a CytoFlex-S flow cytometer (Beckman Coulter, RRID:SCR_019627) and data analyzed by using FlowJo software (BD, RRID:SCR_008520).

#### ADCC reporter bioassays

HCT116/pMIF target cancer cells were seeded in 96-well flat-bottom white plates (Costar) at 1 × 10^4^ cells/well 1 day prior to the assay. Assay was performed according to the manufacturer's protocol for the ADCC reporter bioassay core kit with human FcγRIIIA V158-variant (high responder, Promega, # G7010), or F158-variant (low responder, Promega, # G9790) effector cells by using an effector to target cell ratio of 5:1. Luminescence was measured on a Tecan Infinite M200 PRO (RRID:SCR_019033) microplate reader, and results are presented in relative luminescence units (RLU; mean and range of *n* = 2). EC_50_ values were calculated as described earlier.

#### ADCP reporter bioassay

HCT116/pMIF target cells were seeded in growth medium in 96-well flat-bottom white plates at 1 × 10^4^ cells/well. On the next day, the assay was done according to the manufacturer's protocol for the ADCP reporter bioassay with human FcγRIIA-H131 variant effector cells (Promega, # G9991) by using an effector to target cell ratio of 5:1. Luminescence was measured on a Tecan Infinite M200 PRO (RRID:SCR_019033) microplate reader, and results are presented in RLU (mean and range of *n* = 2). EC_50_ values were calculated as described earlier.

### Characterization of human PBMCs for immune cell populations

PBMCs were isolated from whole blood of healthy volunteers (Red Cross) by gradient centrifugation using Lymphoprep and SepMate 50 tubes according to manufacturer's instructions (StemCell Technologies, RRID:SCR_013642). Cells were frozen immediately after isolation in RPMI1640 medium containing 50% FBS and 10% DMSO at 20–30 × 10^6^ cells per cryovial. In addition, frozen PBMCs of a known FcγRIIIA genotype (healthy volunteer, high responder V158/V158) were purchased from Bio-IVT (www.bioivt.com). For characterization, PBMC aliquots were thawed and a total of 5 × 10^5^ cells per sample were stained with eFluor 780 fixable viability dye (Thermo Fisher Scientific, # 65-0865-14). After blocking Fc receptors with human TruStain FcX solution (Biozym), cells were stained with antibody cocktail (Supplementary Table S2) in PBS containing 2% BSA. After washing, cells were analyzed on the CytoFlex-S cytometer (Beckman Coulter, RRID:SCR_019627). Single stainings with individual antibodies were done to compensate for spectral overlap between the fluorophores. Data were analyzed with FlowJo software (BD, RRID:SCR_008520). Individual immune cell populations (total CD3^+^ T cells, CD4^+^ and CD8^+^ T cells, NK cells, B cells, and monocytes) were defined by the gating strategy shown in Supplementary Fig. S1, and their percentages from the total viable cells were determined (Supplementary Table S3).

### ADCC with human PBMCs

HCT116/HiBiT/pMIF target cancer cells were seeded at 1 × 10^4^ cells/well in 50 μL/well RPMI1640 medium supplemented with 5% ultralow IgG serum (Thermo Fisher Scientific) in 96-well flat-bottom white plates (Costar). On the next day, 50 μL/well of antibody dilutions (0.06–100 nmol/L), followed by 50 μL/well of freshly thawed PBMCs from healthy volunteers were added (5 × 10^5^ cells/well). This results in a ratio of PBMCs to target cells of 50:1. After overnight incubation of target cells with mAbs and PBMCs, target cell lysis was measured by quantifying the release of a HiBiT-tagged HaloTag protein. For this, 10 μL/well of the freshly prepared Nano-Glo HiBiT Extracellular Reagent (Promega, # N2421) was added. After incubation for 3 minutes, luminescence was measured on a Tecan Infinite M200 PRO (RRID:SCR_019033) microplate reader. Results are displayed in RLU (mean and range of *n* = 2). EC_50_ values were calculated as described earlier. The experiment was repeated using PBMCs from 3 different healthy donors.

### Assessment of antibody-dependent cytokine release from human PBMCs in the presence or in the absence of target cells

PBMCs (5 × 10^5^ cells/well) were incubated in 96-well plates with ON203 or C0008 [70, 7, 0.7, 0.07, 0 nmol/L (i.e., medium)] in 150 μL/well RPMI1640 medium supplemented with 5% ultra-low IgG serum (Thermo Fisher Scientific) for 24 hours in the presence or in the absence of HCT116/pMIF target cells (1 × 10^4^ cells/well). Cytokine release was assessed from supernatants using LegendPlex cytometric bead assays (BioLegend, RRID:SCR_001134), for human IL6, MCP-1, TNFα, IFNγ, and IL2 according to the manufacturer's protocol. Samples were read on the CytoFlex-S cytometer (Beckman Coulter, RRID:SCR_019627), and data were analyzed using LegendPlex analysis software Version 8.0 (BioLegend, RRID:SCR_001134).

### Animal studies

These studies were carried out in strict accordance with the recommendations of the GV SOLAS in an Association for Assessment and Accreditation of Laboratory Animal Care International–accredited animal facility. All animal experiments were approved by the Committee on the Ethics of Animal Experiments of the regional council (permit numbers: G17/78, G-18/12, and G-20/163) in Germany.

### Pharmacokinetics and biodistribution

To evaluate pharmacokinetic profiles of C0008, C0083, and C0090, the mAbs were injected intravenously into the tail vein of female BALB/c nude mice (CAnN.Cg-*Foxn1^nu^*/Crl, age 5–7 weeks) at a dose of 10 mg/kg. Twenty microliters of blood were collected 4, 10, 24, 48, and 96 hours after injection by tail vein puncture using K_2_-EDTA–coated capillaries (Minivette POCT). Animals were euthanized after the last sampling by cervical dislocation. Blood was transferred into K_2_-EDTA–coated vials containing 60 μL PBS and after centrifugation, the supernatants (plasma diluted 1:4) were analyzed by a quantitative anti-oxMIF mAb ELISA (final plasma dilution 1:100–1:100,000). The mean concentration of the antibodies per time point was plotted against collection time and half-lives were calculated by a two-phase decay model [SpanFast = (Y0-Plateau)*PercentFast*.01; SpanSlow = (Y0-Plateau)*(100-PercentFast)*.01; Y = Plateau + SpanFast*exp(−KFast*X) + SpanSlow*exp(−KSlow*X); where Y is the antibody concentration, X is the collection time, Kfast and Kslow are the two rate constants, expressed in reciprocal of the X, and Plateau the Y value at infinite times] in GraphPad Prism (RRID:SCR_002798).

Biodistribution (BD) of anti-oxMIF mAbs was investigated in female BALB/c mice (age 9–11 weeks) with subcutaneous CT26 tumors, or in female BALB/c nude mice (CAnN.Cg-*Foxn1^nu^*/Crl, age 5–7 weeks) bearing subcutaneously HCT116 tumors. mAbs were labeled with IRDye 800CW using the IRDye 800CW Protein labeling kit (high MW, LI-COR Biosciences, #929-70020) following the manufacturer's instructions. After the labeling process and prior to injection of labeled antibodies into mice, the protein concentration and labeling efficiency of the IRDye 800CW-labeled antibody was determined using Nanodrop technology (Thermo Fisher Scientific, NanoDrop 2000, RRID:SCR_018042). Mice were dosed on the basis of the protein concentration after labeling. Mice received 3 × 10^5^ CT26 cells or 5 × 10^5^ HCT116 cells in 100 μL PBS unilateral under isoflurane anesthesia. Upon reaching tumor volumes of approximately 150–300 mm^3^, mice were assigned to treatment groups and received a single intravenous dose of 5 mg/kg IRDye 800CW-labeled mAbs. Two mice were used as untreated “no signal” controls. Mice were imaged using LI-COR Pearl Trilogy imaging device at 1, 6, 8, 24, 48, 72, 96, and 168 hours after dosing under isoflurane anesthesia. Animals were euthanized after the last imaging by cervical dislocation. Image analysis was performed in the LI-COR Pearl Trilogy Software to quantify the relative fluorescence per mm² (RFU/mm²) of the antibody in the tumor. A region of interest (ROI) was drawn around the tumor area and the tumor RFU/mm² was calculated using the formula tumor RFU/mm² = RFU ROI tumor/area of ROI. The mean tumor RFU/mm² of the antibodies per timepoint was plotted against imaging time and half-lives were calculated by a one-phase decay model [Y = (Y0 − Plateau)*exp(−K*X) + Plateau]; where Y is the mAb RFU/mm² (Y0, mAb RFU/mm² at time 0), X is the imaging time, K is the rate constant, and Plateau the Y value at infinite times in GraphPad Prism (RRID:SCR_002798).

#### Xenograft efficacy models

Six to 9 weeks old male NMRI nude mice (Crl:NMRI-*Foxn1^nu^*) were subcutaneously injected with 5 × 10^6^ PC3 cells resuspended in 0.1 mL of 1:1 PBS/growth factor–reduced Matrigel (Corning Matrigel GFR Basement Membrane Matrix) under isoflurane anesthesia. Treatment was either initiated 1 day after cell implantation (prophylactic setting, animal randomization, and distribution into experimental groups based on body weight) or when tumor volumes of 70–110 mm³ have been reached (therapeutic setting, animal randomization into experimental groups based on tumor volume). The respective mAbs [resuspended in 25 mmol/L histidine hydrochloride (Sigma-Aldrich), 200 mmol/L Sucrose (Sigma-Aldrich), pH 6.0; or 50 mmol/L histidine hydrochloride (PanReac AppliChem), 50 mmol/L arginine hydrochloride (PanReac AppliChem), pH 5.5, for the prophylactic and therapeutic setting, respectively] were administered intraperitoneally 15 times every second day. Tumor volume (two-dimensional measurement with calipers, *V*_T_ = 0.5 × *L* × *W*²) and body weight were determined twice weekly. At the end of the observation period, animals were euthanized by cervical dislocation and tumors were excised, formalin-fixed, and paraffin-embedded (FFPE) for subsequent IHC analysis.

#### Histology and IHC

Paraffin sections were stained with hematoxylin and eosin (H&E) for morphologic analysis or by IHC analysis for Ki67 (Abcam, ab16667, RRID:AB_302459, dilution 1:200) or CD31 (Dianova, DIA-310, RRID:AB_2631039, dilution 1:25), counterstained by hematoxylin (Shandon Harris Hematoxylin, Thermo Fisher Scientific). Stained slides were scanned using the Pannoramic 250 Flash III digital scanner (3DHistech, RRID:SCR_022184) and representative pictures were obtained with CaseViewer 2.4 (3DHistech, RRID:SCR_017654). For the prophylactic PC3 study, Ki67 and CD31 quantification were performed by a trained pathologist using VIS Image Analysis Software (Visiopharm, RRID:SCR_021711) in the following workflow: (i) drawing of tumor ROI (manually); (ii) detection of necrosis (deep learning classification, project specific, automated); (iii) manual correction of step 2; (iv) quantification of necrosis (automated); for CD31: (v) tumor neovascularization protocol on tumor ROI (excluding necrosis); for CD31 and Ki67: (v) Ki67 protocol (artificial intelligence based) on tumor ROI. In the therapeutic PC3 study, the Ki67-stained tumor slides were scanned by an Olympus Slideview VS200 scanner using a VS-264C camera. Ki67 quantification was done in QuPath version 0.2.3 (RRID:SCR_018257; ref. 36) by a trained pathologist. Tumor area and necrotic tissue were assessed by a machine-learned classifier of random trees and manually corrected if needed.

### Data availability

Data generated in this study are supplied within this article and the Supplementary Data. Raw data are available on request from the corresponding author.

## Results

### Bioengineered second-generation anti-oxMIF mAbs show reduced hydrophobicity and aggregation propensity while maintaining the low nanomolar affinity of the first-generation anti-oxMIF mAb (imalumab)

By *in silico* screening and modeling via proprietary algorithms, we identified residues in the variable domains of imalumab that could potentially contribute to unfavorable properties of this first-generation anti-oxMIF antibody, including high surface hydrophobicity, high aggregation propensity, and an unusually short half-life. First, we expressed and purified 25 mAbs with mutated variable domain sequences and performed *in vitro* screening assays, including ELISA (specificity and apparent affinity to oxMIF), surface plasmon resonance (SPR; affinity to oxMIF), HIC, SEC, and differential scanning fluorimetry, to identify mAbs with optimized physicochemical properties while retaining the affinity and specificity for oxMIF. In addition, we investigated manufacturability criteria such as expression yields and stability. From those data, we were able to select two encouraging variants, C0083 and C0090 (Supplementary Table S1), still possessing the heavy-chain constant region of C0008, a mAb produced in our lab with the identical sequence as imalumab but devoid of the C-terminal lysine. Next, we replaced the heavy-chain constant region of C0083 and C0090 with the heavy-chain constant region of an IgG1/IgG2 hybrid isotype carrying two ADCC/ADCP-enhancing point mutations (S239D/I332E) in the CH2 domain (32, 37). This resulted in our lead candidates, ON103 and ON203, respectively (Supplementary Table S1).

**Figure 1.**
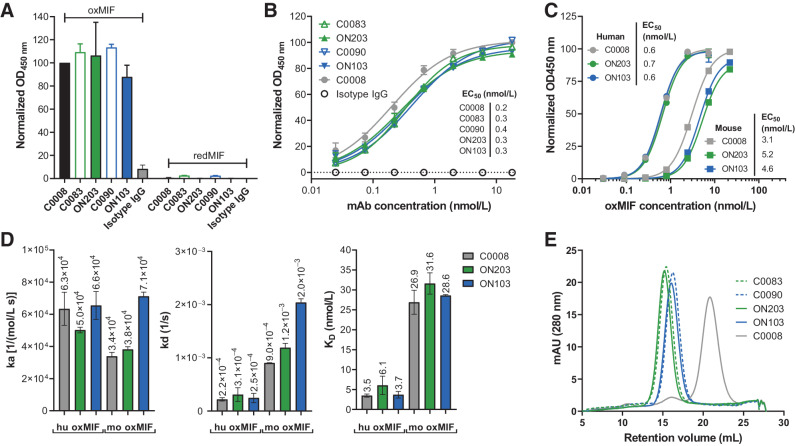
Physicochemical characterization of the mAb variants revealed identical binding properties and reduced hydrophobicity of the bioengineered anti-oxMIF antibodies compared with C0008 (imalumab). **A,** Antibody specificity of oxMIF versus redMIF in solution using 50 ng/mL of an oxMIF surrogate (TNB-MIF) by ELISA. **B,** Antibody apparent affinity to human oxMIF immobilized to an ELISA plate. **C,** Antibody affinity to human and mouse oxMIF in solution (using ProClin300 to induce oxMIF structure) by ELISA. **D,** Real-time binding kinetics of immobilized ON103 and ON203 versus C0008 for soluble human and mouse oxMIF by SPR. Data are represented as mean ± SD of the calculated association constants [ka, 1/(mol/L s)], dissociation constants (kd, 1/second), and equilibrium constants (K_D_, nmol/L) of flow cells 2–4 (after the subtraction of background from the reference cell (FC1). **E,** HIC of bioengineered antibodies compared with imalumab (C0008). EC_50_, effective concentration at 50% signal.

Using three ELISA methods, we (i) investigated the specificity to oxMIF versus redMIF in solution using 50 ng/mL of an oxMIF surrogate [TNB-MIF (12)] (Fig. 1A), (ii) determined the apparent affinity to human oxMIF immobilized to an ELISA plate (Fig. 1B), and (iii) determined the apparent affinity to human and mouse oxMIF in solution [using ProClin300 as described by Thiele and colleagues (10), Fig. 1C]. The antibody candidates retained the specificity to oxMIF, as no binding to redMIF was observed (Fig. 1A). Apparent affinity, including avidity effects, to human oxMIF using the anti-oxMIF mAb ELISA method ranged from 0.2 to 0.4 nmol/L (Fig. 1B). In solution, affinity to human and mouse oxMIF determined by ELISA ranged from 0.6 to 0.7 nmol/L and 3.1–5.2 nmol/L, respectively (Fig. 1C). Affinity to mouse oxMIF was about six to nine times lower compared with human oxMIF for all anti-oxMIF antibodies, as described previously (16). In addition, we determined real-time binding kinetics of ON103 and ON203 versus C0008 for soluble human and mouse oxMIF by SPR revealing low nanomolar affinities of 3.5–6.1 nmol/L and 26.9–31.6 nmol/L, respectively (Fig. 1D; Supplementary Fig. S2), like previously described affinities for imalumab (16), confirming that antibody engineering did not change anti-oxMIF mAb affinities. Epitope mapping using libraries of overlapping 15mer synthetic peptides spanning the whole sequence of human MIF confirmed that ON103 and ON203 bind to the same linear core epitope _54_EPCALC_59_ (Supplementary Fig. S3) as published for BaxB01 (sharing the same Fab regions with imalumab; refs. 12, 16). HIC revealed strongly reduced surface hydrophobicity of the lead candidates in this order, descending from C0083 and ON203 to C0090 and ON103, compared with C0008 as demonstrated by a substantially lower retention volume (Vr 15.2 and 16.1 mL for ON203 and ON103, respectively, vs. 20.8 mL for C0008; Fig. 1E).

### C0083 and C0090 show improved pharmacokinetic profiles and BD compared with C0008

**Figure 2.**
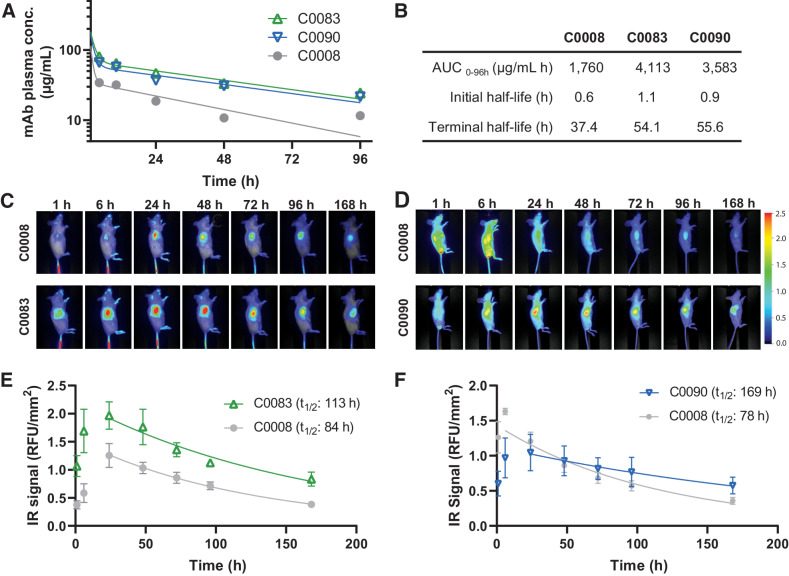
Pharmacokinetics and BD of C0008, C0083, and C0090. **A,** BALB/c nude mice received a single intravenous injection of C0008, C0083, and C0090 mAbs (10 mg/kg), and plasma samples were collected 4, 10, 24, 48, and 96 hours thereafter. The plasma concentration of mAbs was determined by a quantitative anti-oxMIF mAb ELISA. **B,** mAb half-lives were calculated by a two-phase decay model, where plateau was constrained to 0 and Y0 to the theoretical *C*_max_. Total exposure was calculated from the AUC from the two-phase decay model from 0 to 96 hours. **C** and **D,** Tumor penetration and retention of anti-oxMIF mAbs was assessed by infrared *in vivo* imaging. **C,** Female BALB/c mice bearing subcutaneous CT26 syngeneic tumors received a single intravenous injection of 5 mg/kg IRDye 800CW-labeled C0008 or C0083 mAbs. **D,** Female BALB/c nude mice bearing subcutaneous HCT116 xenografts received a single intravenous injection of 5 mg/kg IRDye 800CW-labeled C0008 or C0090 mAbs. **C** and **D,** Mice were imaged at 1, 6–8, 24, 48, 72, 96, and 168 hours after dosing. One representative mouse from each treatment group (*n* = 3) is shown. **E** and **F,** Digital image analysis of tumor ROIs from images **C** and **D** in LI-COR Pearl Trilogy Software and calculation of total exposure of the tumor (AUC) to the anti-oxMIF mAbs and tumor half-life of mAbs using a monoexponential decay model in GraphPad Prism.

It is well established that low solubility of mAbs due to surface hydrophobicity impedes formulation development and may lead to aggregation, poor BD, undesirable pharmacokinetic behavior, and immunogenicity *in vivo* (38–41). Thus, we analyzed the pharmacokinetic profile of our optimized variable domain variants C0083 and C0090 in comparison with the first-generation anti-oxMIF antibody C0008 in BALB/c nude mice. All three antibodies contain an IgG1 constant heavy chain to analyze the impact of the variable domain optimization on mAb pharmacokinetic and BD. Mice received a single intravenous injection of 10 mg/kg anti-oxMIF antibodies, and plasma samples were collected 4, 10, 24, 48, and 96 hours thereafter. Antibody plasma concentrations were determined by a quantitative anti-oxMIF mAb ELISA (Fig. 2A) and the half-lives were calculated by a two-phase decay model (Fig. 2B). Optimization of the variable regions of C0008 resulted in a 1.4- or 1.5-fold longer half-life (terminal half-life: 54.1 hours, 55.6 hours; Fig. 2A) and 2.3- or 2-fold higher overall exposure (AUC: 4,113 μg/mL hour, 3,583 μg/mL hour; Fig. 2B) for C0083 and C0090, respectively, compared with C0008 (terminal half-life: 37.4 hours, AUC: 1,760 μg/mL hour; Fig. 2A and B).

We further investigated BD of C0083 and C0090 compared with C0008. C0083 was evaluated in a syngeneic colon cancer model (BALB/c mice carrying subcutaneous CT26 tumors; Fig. 2C) and C0090 was evaluated in a xenograft model of colon cancer (BALB/c nude mice bearing subcutaneous HCT116 tumors, Fig. 2D). Tumor-bearing animals received a single intravenous injection of 5 mg/kg IRDye 800CW-labeled mAbs with comparable degree of labeling (CT26 syngraft model, C0008: 0.75, C0083: 1.13; HCT116 xenograft model, C0008: 1.29, C0090: 0.83) and were imaged at indicated timepoints; Fig. 2C and D). The intratumoral accumulation of all mAbs peaked at 24 hours and tumor retention was evident up to 7 days. Importantly, by applying digital image analysis and calculation of total exposure of the tumor (AUC) to the anti-oxMIF mAbs and tumor half-life using a monoexponential decay model, we demonstrated that tumor penetration and retention of C0083 (AUC: 222 RFU/mm² hour, half-life: 113 hours; Fig. 2E) and C0090 (AUC: 134 RFU/mm² hour, half-life: 169 hours, Fig. 2F) were considerably improved over C0008 [AUC: 127 RFU/mm² hour (E) and 126 RFU/mm² hour (F), half-life: 84 hours (E) and 78 hours (F); Fig. 2E and F] and are in line with improved pharmacokinetics in wild-type (wt) mice.

In summary, we show that optimization of the variable domains of C0008 resulted in less hydrophobic and less aggregation-prone antibodies, with extended half-life and enhanced tumor penetration and retention in mouse models of colon cancer.

### ON103 and ON203 mAbs with engineered Fc induce potent ADCC and ADCP *in vitro*

Next, the novel anti-oxMIF mAbs ON103 and ON203 were assessed for their ability to initiate ADCC and ADCP as relevant mechanisms of tumor cell eradication in solid tumors in reporter bioassays. First, we assessed oxMIF expression in cancer cell lines PC3 and HCT116 by flow cytometry and found moderate oxMIF expression at the cell surface of both cell lines (Supplementary Fig. S4). To increase assay sensitivity, we produced a HCT116/pMIF cell line stably expressing membrane anchored human MIF on the cell surface (Supplementary Figs. S5 and S6). This allowed us to test the antibodies *in vitro* for their ADCC and ADCP potential. Briefly, Jurkat effector cells engineered to (i) stably express human FcγRIIIA of high responder (V158) and low responder (F158) genotypes to assess ADCC or (ii) stably express human FcγRIIA (H131) to assess ADCP were incubated with serial dilutions of mAbs in the presence of HCT116/pMIF target cells.

**Figure 3.**
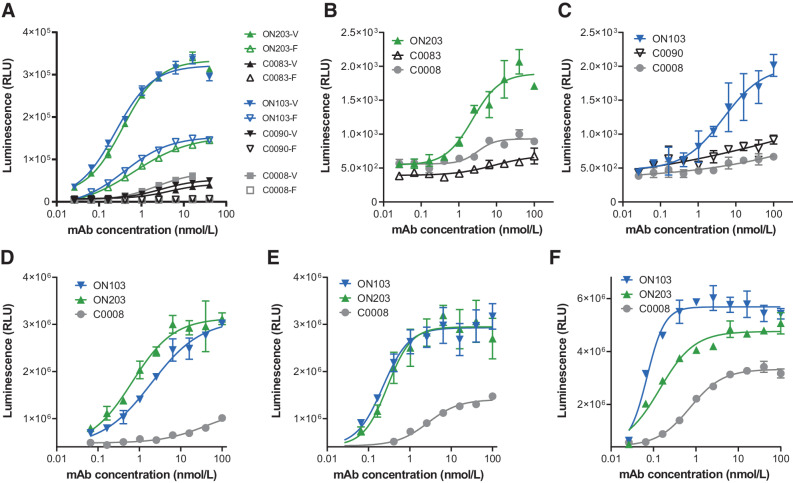
ON103 and ON203 mAbs with engineered Fc induce potent ADCC and ADCP. **A,** ON103 and ON203 trigger potent effector cell activation in ADCC reporter bioassays. Serial dilutions of mAbs were incubated for 6 hours with HCT116/pMIF target cells and Jurkat effector cells engineered to stably express human FcγRIIIA of high responder (V158) genotype, or of low responder (F158) genotype, and an NFAT response element driving expression of firefly luciferase. Effector cell activation was quantified through luciferase (RLU) produced as the result of NFAT pathway activation in effector cells (mean and range of *n* = 2). ON203 (**B**) and ON103 (**C**) induce ADCP in an ADCP reporter bioassay. Serial dilutions of mAbs were incubated for 6 hours with target cells (HCT116/pMIF) and Jurkat effector cells engineered to stably express human FcγRIIA (high responder H131 genotype), and an NFAT response element driving expression of firefly luciferase. Effector cell activation was quantified as described in **A** (mean and range of *n* = 2). **D–F,** ON103 and ON203 induce significant target cell lysis in an ADCC assay with human PBMCs. Target cells HCT116/HiBiT/pMIF were incubated with serial dilutions of mAbs and with isolated PBMCs from 3 different healthy human donors at the ratio of PBMCs to target cells of 50:1. PBMCs genotyped having V158/V158 high responder genotype of FcγRIIIA and 7% NK cells (**D**); nongenotyped PBMCs, 22.5% NK cells (**E**); nongenotyped PBMCs, 22.1% NK cells (**F**). After 24 hours, target cell lysis was measured by using NanoGlo HiBiT extracellular detection. Results are expressed in RLU (mean and range of *n* = 2). NFAT, nuclear factor of activated T cells.

As predicted, the second-generation anti-oxMIF mAbs ON103 and ON203 induced strong activation of effector cells expressing either the high responder (EC_50_: 0.2–0.4 nmol/L, V158) or the low responder (EC_50_: 0.4–0.7 nmol/L, F158) genotype of FcγRIIIA with similar EC_50_s in the low nanomolar range (Fig. 3A). This is an important feature due to the high prevalence of homozygous (F/F) and heterozygous (F/V) genotype (35%–40% and 40%–45%, respectively) in the human population worldwide (22, 25, 30). The anti-oxMIF mAbs carrying an IgG1 wt heavy-chain constant region (C0008, C0083, and C0090) were not able to trigger any detectable activation of low responder genotype (F158) effector cells and only induced a modest activation of high responder genotype (V158) effector cells with >10-fold higher EC_50_ values (2.3–4.5 nmol/L) compared with ON103 and ON203 (Fig. 3A). In addition, ON103 and ON203 induced substantial activation of reporter cells in an ADCP reporter bioassay, whereas no reasonable activation of reporter cells was detected for C0008, C0083, and C0090 carrying a wt IgG1 Fc (Fig. 3B and C).

Furthermore, ON103 and ON203 were analyzed for the ability to induce target dependent tumor cell lysis in a human PBMC-mediated ADCC assay compared with C0008 (Fig. 3D–F). We generated highly responsive reporter target cells by stably expressing intracellular HiBiT-tagged HaloTag protein in HCT116/pMIF cells (termed HCT116/HiBiT/pMIF; Supplementary Fig. S6), where cytotoxicity is measured by the release of HiBiT-tagged HaloTag upon target cell lysis. Both ON103 and ON203 were comparably potent in triggering ADCC (EC_50_, ON103: 0.07–2.0 nmol/L; ON203: 0.1–0.7 nmol/L), while C0008 showed approximately 10-fold lower target cell lysis induced by human PBMCs (EC_50_, 0.7–39.2 nmol/L) depending on PBMC donor (donor characterization, see Supplementary Fig. S1 and Supplementary Tables S2 and S3).

### ON203 leads to specific antibody-dependent cytokine release from immune cells

**Figure 4.**
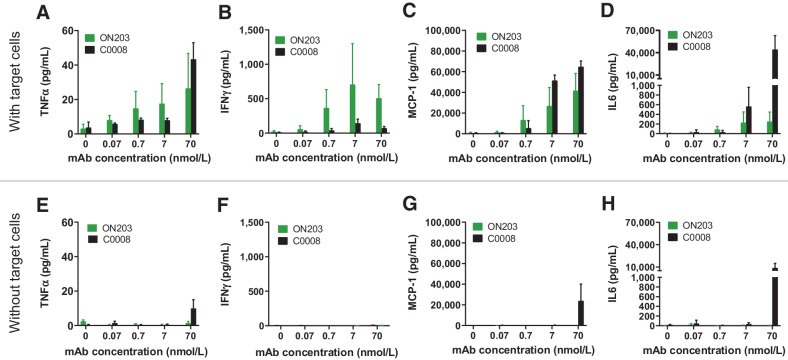
ON203 shows target-dependent cytokine release from PBMCs and reduced unspecific cytokine release compared with C0008 in an ADCR assay. ON203 and C0008 were incubated with human PBMCs for 24 hours in the presence (**A–D**) or in the absence (**E–H**) of HCT116/pMIF target cancer cells, and supernatants were analyzed for the release of TNFα (**A** and **E**), IFNγ (**B** and **F**), MCP-1 (**C** and **G**), and IL6 (**D** and **H**) using LegendPlex cytometric bead assays (BioLegend). Cytokine concentrations in supernatants (pg/mL) were plotted against mAb concentrations (nmol/L) in GraphPad Prism. Values represent mean ± SEM of *n* = 3 (PBMCs from 3 healthy donors). ADCR, antibody-dependent cytokine release; PBMC, peripheral blood mononuclear cell; SEM, standard error of mean.

Antibody-dependent cytokine release (ADCR) can be triggered nonspecifically by a variety of factors such as mAb aggregation (34), but can also be associated with the mode of action (ADCC/ADCP), thus contributing to the therapeutic efficacy. To gain more insight into the differences observed in ADCC potency between ON203 and C0008, an ADCR assay was performed with human PBMCs in the presence of target cancer cells and anti-oxMIF mAbs. In a control experiment PBMCs were incubated with the anti-oxMIF mAbs but without target cancer cells. Supernatants were analyzed in multiplex cytometric bead assays using a panel of proinflammatory cytokines (IL2, IFNγ, ΤΝFα, MCP-1, and IL6). These cytokines are known to be associated with effector–target or effector–effector cellular interactions. The release of cytokines after 24 hours incubation of PBMCs (effector cells) with or without HCT116/pMIF tumor cells (target cells) and various concentrations of C0008 and ON203 (70, 7, 0.7, 0.07 nmol/L) is shown in Fig. 4. For both mAbs, a significant dose-dependent release of TNFα during ADCR was found (Fig. 4A). When comparing the dose dependency for the two mAbs in ADCR, the ADCC-enhanced mAb ON203 resulted in about 2-fold higher TNFα release at lower concentrations when compared with the parental mAb C0008 (Fig. 4A). Most importantly, whereas almost no IFNγ was detected after incubation with C0008 (even at the highest concentrations), substantial levels of IFNγ were measured after incubation with ON203 (Fig. 4B). This correlates well with the differences observed in ADCC activity (Fig. 3D-F). Interestingly, although both mAbs showed a dose-dependent release of MCP-1 and IL6, C0008 resulted in a 1.6- to 170-fold increased cytokine release, respectively, compared with ON203 (Fig. 4C and D), which can be at least partially explained by a target-unrelated cytokine release in case of C0008 (Fig. 4G and H). No measurable levels of IL2 were detected for either of the two mAbs under investigation. Despite the increased affinity of ON203 to activatory FcγRs, no measurable levels of cytokines were detected in supernatants without target tumor cells (Fig. 4E–H). In contrast, C0008 at the highest concentration resulted in a substantial release of TNFα, MCP-1, and IL6 from PBMCs without target cells (Fig. 4E–H) at levels 3- to 5-fold lower than in the presence of target cancer cells (Fig. 4A–D).

In summary, both mAbs ON203 and ON103 showed very similar physicochemical characteristics, pharmacokinetic/BD profile and *in vitro* efficacy. For the following *in vivo* studies, we have prioritized ON203 over ON103 because it is less hydrophobic and closer to the respective human germline (Vk1-39) which is well known to reduce the potential of immunogenicity.

### ON203 significantly inhibits the growth of human PC3 prostate cancer mouse xenografts in both prophylactic and therapeutic settings

**Figure 5.**
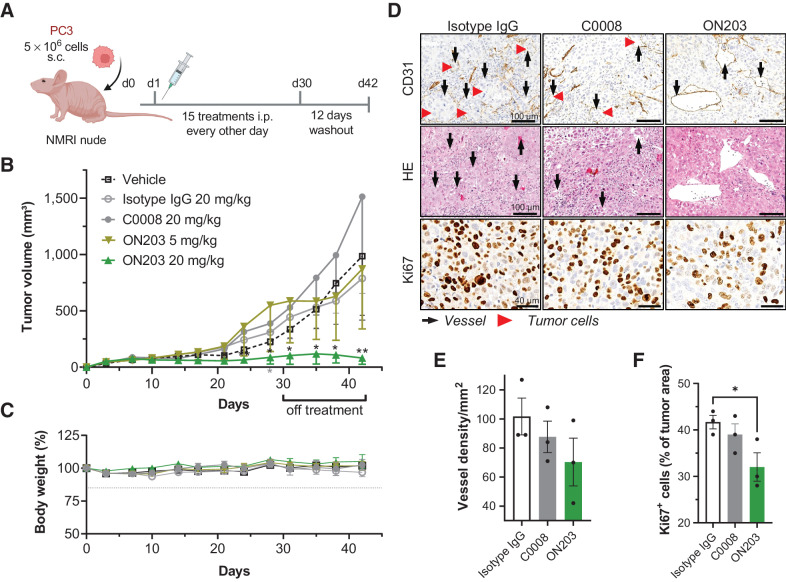
ON203 is efficacious in a prophylactic prostate cancer xenograft model. **A,** Schematic presentation of the prophylactic prostate cancer xenograft model created with www.biorender.com. **B,** Tumor volume of subcutaneous PC3 tumor-bearing mice treated 15 times every other day intraperitoneally with vehicle, 20 mg/kg isotype IgG control antibody, 20 mg/kg C0008, 5 mg/kg ON203, or 20 mg/kg ON203 followed for 42 days after randomization (*n* = 9/treatment group). Median tumor volumes with interquartile ranges are shown. Levels of significance were calculated for all groups by ANOVA with Dunnett multiple comparisons test on log-transformed data; ON203 20 mg/kg versus vehicle + isotype IgG 20 mg/kg (black stars), ON203 20 mg/kg versus C0008 20 mg/kg (gray star) *, *P* < 0.05; **, *P* < 0.01. **C,** Relative change in animal body weight (%) from day 0 (*n* = 9/treatment group, mean ± SEM). **D,** Representative immunostaining images of 20 mg/kg isotype IgG-, C0008-, or ON203-treated PC3 tumors; scale bar, 100 μm (CD31 and H&E) or 40 μm (Ki67). Top: CD31 immunostaining (red arrows highlight intravascular tumor cells, and black arrows mark CD31-positive blood vessels); middle: H&E; bottom: Ki67 immunostaining. **E,** Vessel density in non-necrotic areas quantified on CD31-stained IHC slides from 20 mg/kg isotype IgG control-, C0008-, or ON203-treated tumors (*n* = 3/treatment group, mean ± SEM). **F,** Percentage of Ki67-positive cells quantified on Ki67-positive stained IHC slides from 20 mg/kg isotype IgG control-, C0008-, or ON203-treated tumors (*n* = 3/treatment group, mean ± SEM). Levels of significance were calculated by one-sided Student *t* test, *, *P* <0.05. i.p., intraperitoneal; SEM, standard error of mean.

**Figure 6.**
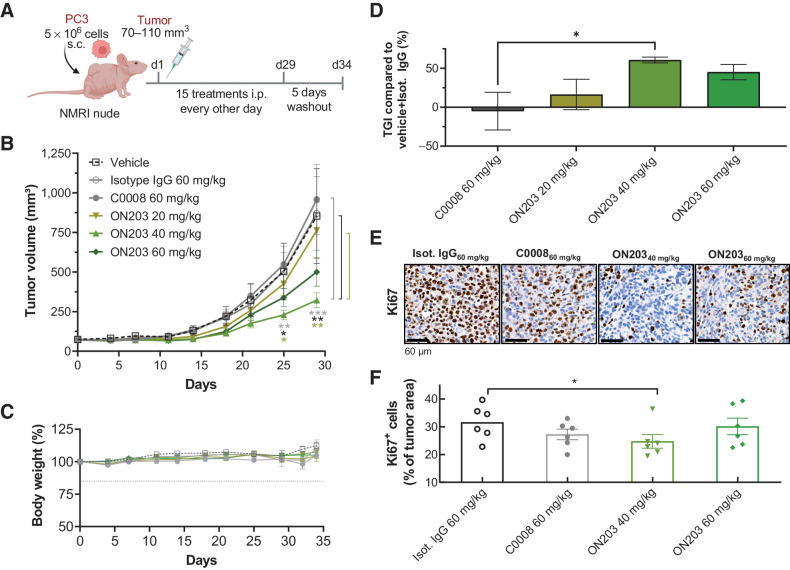
ON203 inhibits tumor growth in a therapeutic prostate cancer xenograft model. **A,** Schematic presentation of the therapeutic prostate cancer xenograft model created with www.biorender.com. **B,** Tumor volume of subcutaneous PC3 tumor-bearing mice treated 15 times every other day intraperitoneally with vehicle, 60 mg/kg isotype IgG control antibody, 60 mg/kg C0008, or 20, 40, or 60 mg/kg ON203 followed for 34 days after randomization (*n* = 7–9/treatment group). Mean tumor volumes ±SEM are shown. Only data up to day 29 could be evaluated, because from this point on several animals had to be sacrificed because of excessive tumor growth. Levels of significance were calculated for all groups by repeated measures two-way ANOVA with Dunnett multiple comparison correction on log-transformed data; ON203 versus C0008 (gray stars); ON203 versus vehicle + isotype IgG (black stars); ON203 40 mg/kg versus ON203 20 mg/kg (green stars) *, *P* ≤0.05; **, *P* < 0.01; ***, *P* < 0.001. **C,** Relative body weight change of animals in percent to day 0 (*n* = 7–9/treatment group, mean ± SEM). **D,** Mean tumor growth inhibition (TGI, mean ± SEM) by C0008 (60 mg/kg) versus ON203 (20, 40, or 60 mg/kg) calculated in percentage relative to the mean tumor volume from vehicle plus isotype IgG. Levels of significance were calculated by ANOVA with Dunnett multiple comparisons test, *, *P* < 0.05. **E,** Representative images of Ki67 immunostaining of 60 mg/kg isotype IgG, 60 mg/kg C0008, or 40 mg/kg or 60 mg/kg ON203-treated PC3 tumors; scale bar, 60 μm. **F,** Percentage of Ki67-positive cells on IHC slides from 60 mg/kg isotype IgG, 60 mg/kg C0008, or 40 and 60 mg/kg ON203-treated tumors (*n* = 6/treatment group, mean ± SEM). Levels of significance were calculated by one-sided Student *t* test on log-transformed data, *, *P* ≤0.05. i.p., intraperitoneal; s.c., subcutaneous; SEM, standard error of mean; TGI, tumor growth inhibition.

Finally, we assessed whether the *in vitro* potency of the second-generation anti-oxMIF mAb ON203 translates into *in vivo* efficacy. The antitumorigenic activity of ON203 was investigated in male NMRI nude mice subcutaneously injected with the human prostate cancer cell line PC3. Animals were treated 15 times every other day with ON203, control antibodies (C0008 and isotype IgG1), or vehicle control in two different settings: (i) prophylactic (treatment initiated 1 day after tumor cell injection; Fig. 5A) or (ii) therapeutic (treatment initiated when tumor volumes reached 70–110 mm^3^; Fig. 6A). Treatment in the prophylactic setting was followed by a 12-day washout period. The efficacy of ON203 was compared with an isotype IgG1, C0008, and vehicle.

In the prophylactic setting, 20 mg/kg ON203 significantly inhibited tumor growth, whereas 5 mg/kg did not show a considerable difference compared with vehicle, isotype IgG, or C0008 (Fig. 5B). In this setting, C0008 at 20 mg/kg did not show any signs of efficacy. Importantly, a durable response was observed for ON203 during the observation period of up to 12 days posttreatment. No acute signs of toxicity were observed and the body weight of mice from all treatment groups did not change over time (Fig. 5C). At the end of the observation period, tumors were collected and FFPE sections were stained with H&E and for Ki67 and CD31 by IHC (Fig. 5D). The smaller tumors in 20 mg/kg ON203-treated mice correspond to reduced proliferation of tumor cells confirmed by significantly reduced number of Ki67-positive cells (Fig. 5D, bottom and Fig. 5F). The intravasation of PC3 tumor cells into the vessels seems to be impaired upon ON203 treatment as the lumen of CD31-stained endothelial cells (vessels) lack tumor cells (Fig. 5D, top and middle). Furthermore, ON203 reduced the vascularization of the subcutaneous tumors in comparison with C0008 and isotype IgG control quantified by lower vessel density (Fig. 5E), albeit not statistically significant.

In a therapeutic setting, the efficacy of ON203 was determined by treating mice bearing preestablished PC3 tumors with 20, 40, or 60 mg/kg ON203, control antibodies (C0008 and isotype IgG at 60 mg/kg), or vehicle (Fig. 6B). The increased dosages were well tolerated as the body weight of animals in all treatment groups did not change (Fig. 6C). Best responses were observed upon treatment with 40 mg/kg ON203 resulting in significantly smaller tumors compared with C0008, vehicle, and IgG1 isotype (Fig. 6B). On the last treatment day (day 29), 40 and 60 mg/kg ON203 reduced the mean tumor volume by 61% and 45%, respectively, whereas the lower dose of ON203 (20 mg/kg) reduced the mean tumor volume by 10% in comparison with vehicle-treated or isotype IgG-treated animals and C0008 (Fig. 6B and D). Again, Ki67 staining confirmed reduced tumor cell proliferation upon treatment with 40 and 60 mg/kg ON203 (Fig. 6E and F). Also in this setting, C0008 at 60 mg/kg did not show any signs of efficacy.

In summary, our optimization strategy to generate second-generation anti-oxMIF antibodies resulted in superior *in vivo* efficacy of ON203 in human prostate cancer xenograft models in comparison with the first-generation anti-oxMIF antibody C0008.

## Discussion

MIF is involved in several proinflammatory, antiapoptotic, proangiogenic, and proproliferative signaling cascades (4–6). Preclinical and clinical studies have demonstrated that MIF is overexpressed in many different types of human cancers (i.e., lung, ovarian, colorectal, pancreatic, breast, gastric, bladder, head and neck cancer, neuroblastoma, melanoma, and acute myeloid leukemia) and correlates with tumor aggressiveness (8, 11). Because MIF is constitutively expressed and present at reasonably high levels in circulation (∼6 ng/mL; ref. 10) and tissues of healthy subjects (10, 11), it can be considered an inappropriate target for pharmaceutical intervention due to the risk of interfering with its nonpathologic functions (13). However, the redox-dependent conformational isoform, oxMIF, is a promising tumor target due to its selective occurrence in tumor lesions and at inflammatory sites (10, 11). Clinical evaluation of anti-oxMIF mAb imalumab, which selectively binds oxMIF but not redMIF, showed, despite its unusually short half-life and lack of ADCC induction, some antitumor activity in patients (17, 18). Our analysis of imalumab's physicochemical properties revealed a variable domain–mediated surface hydrophobicity of the molecule. In the work presented here, we successfully optimized the first-generation anti-oxMIF mAb (imalumab) to create second-generation anti-oxMIF antibodies. When compared with imalumab (or more precisely, mAb C0008, which has the same sequence as imalumab but lacks the C-terminal lysine), these antibodies demonstrated lower aggregation potential and surface hydrophobicity, augmented effector function *in vitro*, improved *in vitro* safety, longer *in vivo* half-life, higher tumor accumulation and retention, and superior antitumor activity *in vivo*.

Hydrophobicity is well known to be involved in protein aggregation (38). Our optimization strategy identified a combination of mutations in the variable regions (variable light chain—VL: F49Y/A51G/W93F; variable heavy chain—VH: L5Q/W97Y) conferring a remarkable reduction of surface hydrophobicity and, consequently, aggregation to the second-generation anti-oxMIF mAbs in comparison with imalumab. Interestingly, from this combination, the two mutations—VL: W93F and VH: W97Y—are located in the third complementarity-determining region (CDR3), which is well known to be the most critical region due to its essential role for antigen binding (38). Importantly, our second-generation anti-oxMIF mAbs carrying these mutations retain their low nanomolar affinity and specificity to oxMIF, by binding to the same linear epitope as imalumab.

It was reported that antibody variable regions influence mAb pharmacokinetics (41). Low solubility of mAbs due to surface hydrophobicity may result in poor BD and undesirable pharmacokinetic behavior (38, 39). In agreement with these findings, reduction of hydrophobicity and aggregation potential by optimizing imalumab-variable regions resulted in a 1.4- to 1.5-fold longer half-life, 2- to 2.3-fold higher overall exposure, and tumor accumulation, as evidenced for C0083 and C0090 in pharmacokinetic/BD studies in mice, whereas these antibodies contain optimized variable domains but share the same constant regions with imalumab.

Mutating the antibody's Fc region to increase its affinity to the activatory human Fcγ receptors, particularly FcγRIIIA and FcγRIIA, represents a clinically validated option to increase ADCC and ADCP, both having a crucial role in tumor elimination by immune cells (20, 26). Equipping our second-generation anti-oxMIF mAbs ON103 and ON203 with a constant heavy chain, which includes two ADCC/ADCP-enhancing point mutations (S239D/I332E) in the CH2 domain (32) and isotypic modifications (IgG1/IgG2 hybrid; ref. 37), resulted in a marked increase of *in vitro* ADCC/ADCP over imalumab. Like the two recently approved ADCC-enhanced mAbs margetuximab (anti-Her2) and tafasitamab (anti-CD19; refs. 22, 32), ON203 and ON103 demonstrated strongly elevated ADCC for both allotypes of the FcγRIIIA (F158, low responder and V158, high responder). Given the predominance of F158 in the human population worldwide (22, 25, 30), increased efficiency in low responder individuals will be an important clinical parameter for our mAb candidate(s).

Antibody-mediated bridging between FcγRIIIA/FcγRIIA on NK cells/phagocytes and target antigen on the tumor cells can trigger the release of IFNγ, TNFα, IL6, and MCP-1 during the ADCC reaction (42) and NK cell–secreted IFNγ and TNFα were shown to potentiate the NK-mediated target cell lysis (43). Nordstrom and colleagues reported the release of IL6 (∼350–400 pg/mL), TNFα, and IFNγ upon the treatment of PBMCs with margetuximab (MGAH22, 10 μg/mL or ∼70 nmol/L) in the presence of target antigen (Her2; ref. 22). In agreement with this study, we observed a dose-dependent ADCR at similar levels of IL6 (∼200 pg/mL at 70 nmol/L), IFNγ, TNFα, as well as MCP-1, upon the treatment of PBMCs with our ADCC-enhanced lead candidate ON203 in the presence of target cells only. Cytokines levels of IFNγ  and ΤΝFα, being a signature of NK activation, were markedly higher for ADCC-enhanced mAb ON203 than for C0008. Thus, ON203-induced ADCR correlated with its ADCC activity and argues for a mode-of-action–specific cytokine release potentiating mAb efficacy. Noteworthy, C0008 at the highest concentration resulted in a substantial release of IL6, TNFα, and MCP-1 from PBMCs without target cells, whereas ON203 did not result in any substantial cytokine release at any concentration tested. We believe this is a result of the reduced hydrophobicity of ON203, which is assumed to minimize nonspecific/off-target binding compared with C0008. This potentially leads to an even more favorable safety profile in humans, even though imalumab (C0008) already showed an acceptable safety profile with a MTD of 37.5 mg/kg in a phase I study (NCT01765790; ref. 18).

In a prophylactic PC3 prostate cancer xenograft model, treatment with the second-generation anti-oxMIF antibody ON203 resulted in superior tumor growth inhibition compared with C0008 (imalumab) administered at the same dose. Importantly, in this prophylactic setting, the tumors did not regrow in the off-treatment observation period, indicative of ON203-induced tumor cell eradication. The significant decrease of tumor growth and cell proliferation (evidenced by the significantly reduced number of Ki67-positive cells) by ON203 in the prophylactic PC3 xenograft model is in agreement with previous studies where first-generation anti-oxMIF antibodies (BaxG03, BaxB01, and BaxM159) reduced prostate cancer cell proliferation and survival by inhibiting MIF-induced activation of ERK1/2- and AKT-signaling pathways *in vitro* and lowered the tumor burden and proliferation rate (Ki67-positive cell counts) *in vivo* (17), albeit an abolishment of tumor growth at 20 mg/kg, as demonstrated by ON203, was not observed in the previous studies.

Evaluation of anti-oxMIF antibody treatment in mice with preestablished PC3 tumors resulted in favorable responses when mice were treated with ON203, evidenced by a significant tumor growth inhibition of approximately 60%. Again, C0008 did not show any signs of efficacy in this model. The efficacy of first-generation anti-oxMIF mAbs in an established mouse tumor model has never been reported. Comparison of our second-generation antibodies to C0008 demonstrates the advantageous effects of our antibody optimization strategy and suggests substantial engagement of effector cells. However, in humans, ADCC is induced by NK cells expressing FcγRIIIA (44), whereas in mice, macrophages expressing the FcγRIV (mouse ortholog of human FcγRIIIA) mediate antibody-dependent effector functions (30). Therefore, the results of our animal studies might still underestimate the efficacy of ON203 in a humanized or human setting.

Consistent with the described role of MIF as a cytokine promoting tumorigenesis via increasing tumor-promoting angiogenesis and vascular permeability (45–48), we observed reduced neoangiogenesis and reduced intravasation of tumor cells upon treatment with ON203. Generally, our *in vitro* and *in vivo* findings substantiate that the pathologic properties attributed to MIF are evidently modified by anti-oxMIF antibodies. Therefore, we speculate that pathologic properties of MIF can be attributed to oxMIF, which reflects a druggable isoform of MIF in cancer (13).

There is substantial scientific evidence that cancer cells utilize secreted MIF to evade immune cell–mediated antitumor responses in the tumor microenvironment (49). MIF is secreted by exhausted T cells in the tumor which supports the polarization of macrophages to immunosuppressive phenotypes (50). Furthermore, knockout (by CRISPR-Cas9) or knockdown (by short hairpin RNAs) of MIF reversed immune-suppressive myeloid-derived suppressor cells to an immunostimulatory dendritic cell–like phenotype and favors the accrual of macrophages displaying inflammatory factors and antigen-presenting profiles (51, 52). Anti-oxMIF antibodies could amplify responses of innate cells in the tumor microenvironment and act synergistically with checkpoint inhibitors that primarily address the modulation of the T-cell axis.

In conclusion, our results support the continued development of anti-oxMIF mAb ON203 as a therapy for patients with solid tumors. The bioengineering efforts described here improve the manufacturability, half-life, safety, and efficacy of second-generation oxMIF-specific antibodies compared with imalumab. Further efforts are warranted to validate ON203 in clinical trials as well as identify the optimal combination with standard-of-care regimens, such as chemotherapy, kinase inhibitors, antiangiogenesis drugs, and checkpoint inhibitors.

## Supplementary Material

Supplementary Tables & Figures Includes Supplementary Tables S1-S3 and Supplementary Figures S1-S6
