# Supplementary material for: Preclinical Evaluation of ON203, A Novel Bioengineered mAb Targeting Oxidized Macrophage Migration Inhibitory Factor as an Anticancer Therapeutic
Source: Mol Cancer Ther. 2023 Apr 17;22(5):555–69. doi: 10.1158/1535-7163.MCT-22-0676 (PMC10157364; doi:10.1158/1535-7163.MCT-22-0676)
Supplement: Supplementary Tables & Figures — Includes Supplementary Tables S1-S3 and Supplementary Figures S1-S6 [file mct-22-0676_supplementary_tables__figures_suppsft.pdf]

Preclinical evaluation of ON203, a novel bioengineered monoclonal antibody targeting oxidized macrophage migration inhibitory factor as an anti-cancer therapeutic

Gregor Rossmueller[1§], Irina Mirkina[1§], Barbara Maurer[1], Verena Hoeld[1], Julia Mayer[1], Michael Thiele[1], Randolf J. Kerschbaumer[1], Alexander Schinagl[1]

[1]OncoOne Research & Development GmbH, Karl-Farkas-Gasse 22, A-1030 Vienna, Austria.
[§]equal contribution.

SUPPLEMENTARY TABLES & DATA

Supplementary Table S1

MAb ID	LC mutations	HC mutations
**C0008**	VL: wt<br>CL: wt	VH: wt<br>CH: del477K
**C0083** (1)	VL: F49Y/A51G/W93F<br>CL: wt	VH: L5Q/W97Y<br>CH: del477K
**C0090** (1)	VL:F49Y/A51G/W93F/M30L/P80S<br>CL: wt	VH: L5Q/W97Y<br>CH: del477K
**ON103** (1)	VL: F49Y/A51G/W93F/M30L/P80S<br>CL: wt	VH: L5Q/W97Y<br>CH:*R214K*/S239D/K274Q/Y296F/Y300F/ L309V/I332E/A339T/V397M/del477K (2,3)
**ON203** (1)	VL: F49Y/A51G/W93F<br>CL: wt	VH: L5Q/W97Y<br>CH:*R214K*/S239D/K274Q/Y296F/Y300F/ L309V/I332E/A339T/V397M/del477K (2,3)

Supplementary Table S2. **Mutations of second-generation anti-oxMIF mAbs C0083, C0090, ON103, and ON203 compared to imalumab (C0008)** (1). C0008, corresponds to the sequence of imalumab devoid of the C-terminal lysine produced in our lab, and "wt" refers to the respective sequence of imalumab (Bax69, GenBank JB325049.1 (LC), GenBank JB325055.1 (HC). Residues in the variable domains are numbered according to Kabat (4), whereas mutations in the constant regions are numbered according to EU numbering (5); and *italicized* mutations are allotype specific. CH, constant region of the heavy chain; CL, constant region of the light chain; HC, heavy chain; LC, light chain; mAb, monoclonal antibody; VH, variable region of the heavy chain; VL, variable region of the light chain; wt, wild type.

Supplementary Table S2. Flow cytometry panel of antibodies for human PBMC characterization for immune cell population.

	Laser (nm)	BP filter (nm)	Provider	Catalog/RRID	Clone
**eFluor™780 Fixable Viability Dye**	638 (red)	780/60	Invitrogen	65-0865-14	-
**CD3-BV421**	405 (violet)	450/45	BioLegend	300434/RRID:AB_10962690	UCHT1
**CD4-APC**	638 (red)	660/10	BioLegend	300537/RRID:AB_2562051	RPA-T4
**CD8-FITC**	488 (blue)	525/40	BioLegend	301050/RRID:AB_2562055	RPA-T8
**CD14-PE/Dazzle594**	561 (yellow)	610/20	BioLegend	301852/RRID:AB_2629576	M5E2
**CD19-PerCP/Cy5.5**	488 (blue)	690/50	BioLegend	363016/RRID:AB_2564207	SJ25C1
**CD56-PE**	561 (yellow)	585/42	BioLegend	318306/RRID:AB_604101	HCD56

Supplementary Figure S1

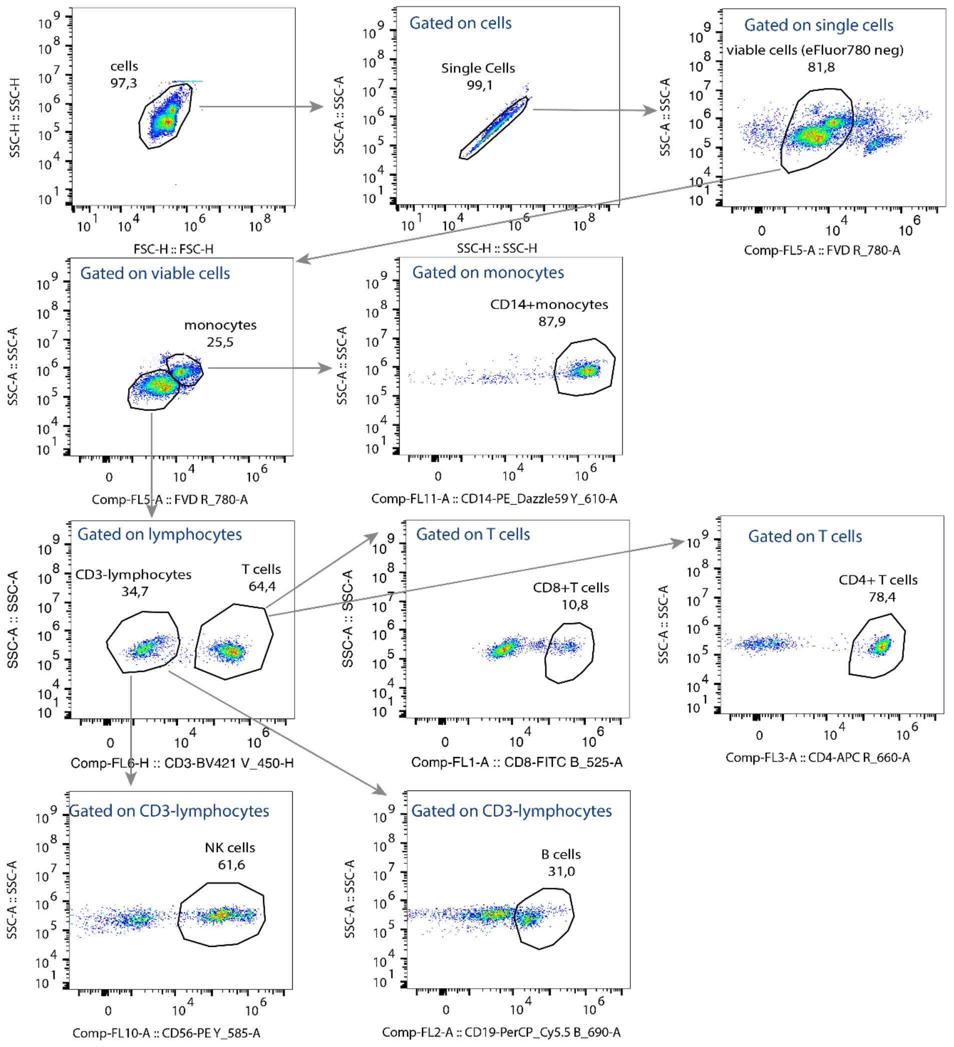

Supplementary Figure S1. Gating strategy to define individual immune cell populations in human PBMCs. Individual immune cell populations (total T cells (CD3+), CD4+ and CD8+ T cells, NK cells, B cells, and monocytes) were defined using the shown gating strategy, and their percentage from the total viable cells (eFluor™780 negative) were determined (**Supplementary Table S3**).

Supplementary Table S3. PBMC characterization for immune cell populations.

PBMCs	CD3+ T cells	CD4+ T cells	CD8+ T cells	B cells	NK cells	Monocytes
**Donor #13 (Red Cross/FcγRIIIA genotype unknown)**	53.9%	44.8%	7.7%	5.3%	22.1%	9.8%
**Donor #14 (RedCross/FcγRIIIA genotype unknown)**	58.9%	42.8%	12.7%	2.1%	22.5%	8.8%
**Donor # CC00087 (Bio-IVT/FcγRIIIA genotype V158/V158)**	50.4%	30.4%	16.0%	7.4%	7.0%	24.7%

Supplementary Figure S2

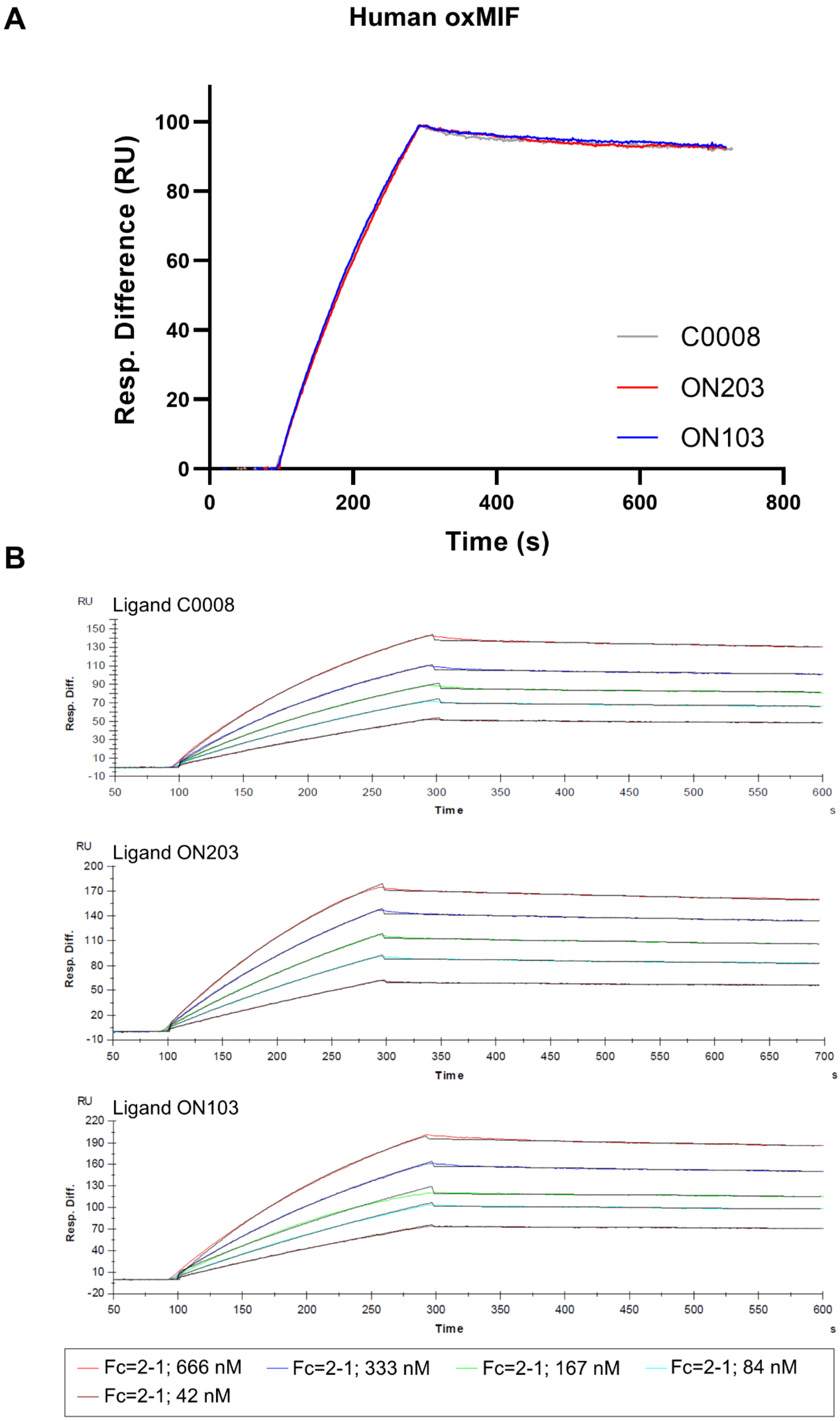

Supplementary Figure S2 continued

C

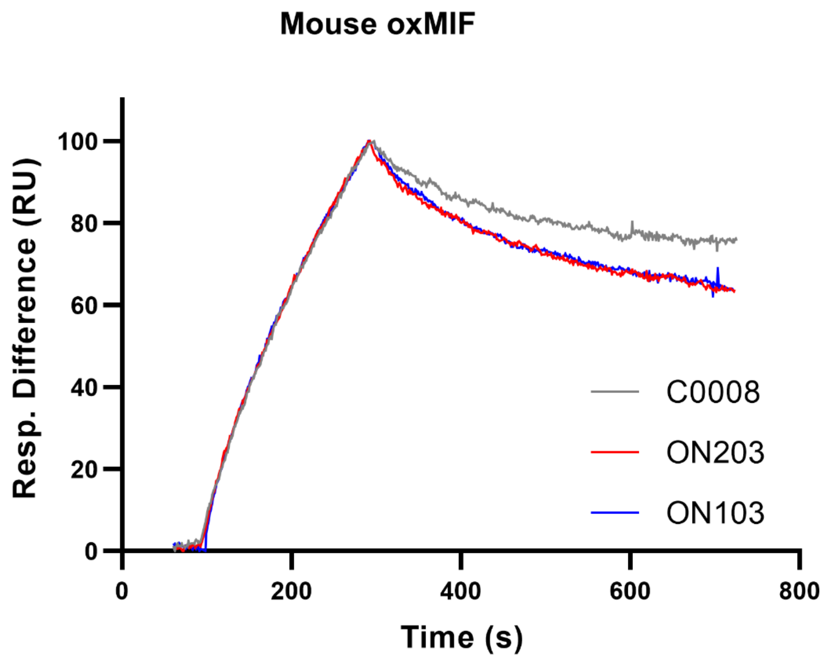

D

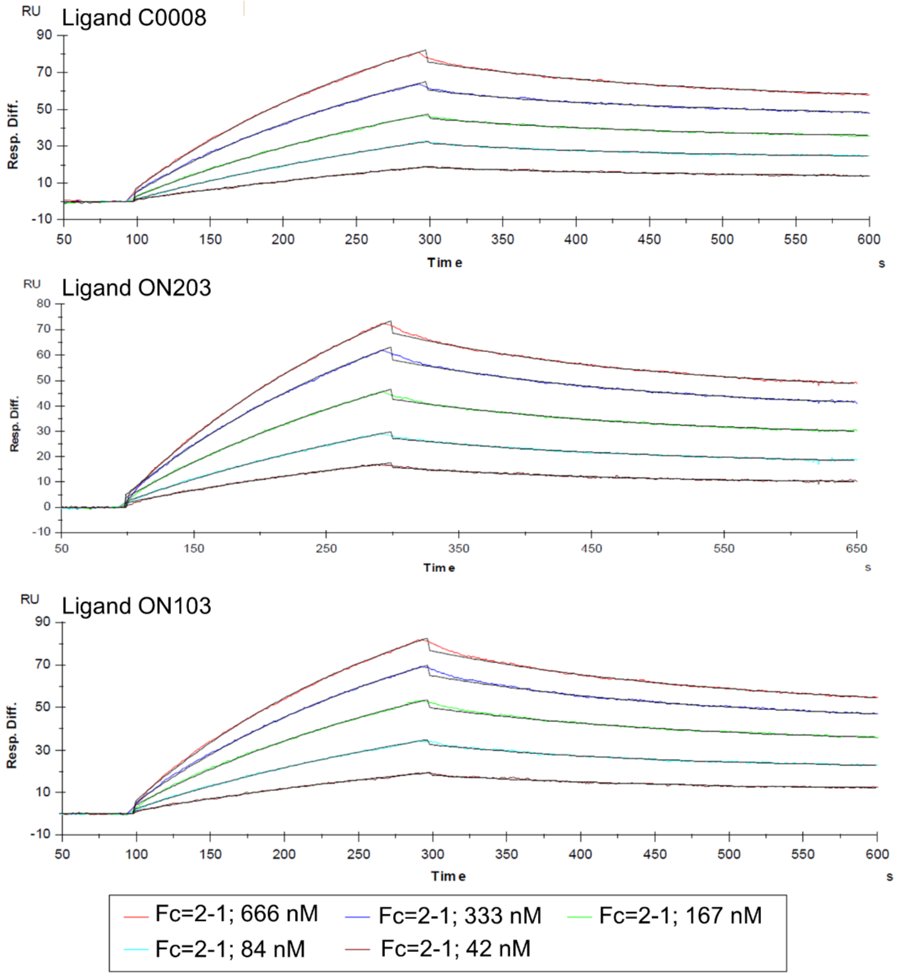

Supplementary Figure S2. Comparison of human (A-B) and mouse (C-D) oxMIF SPR sensorgrams. A & C. In order to allow comparative visualization of differences in association and dissociation parts of the sensorgrams generated with the test antibodies, 167nM sensorgrams were y-normalized for RUmin = 0 RU and RUmax = 100 RU. Thus, differences across all parts of the sensorgrams are visible as % deviation from mAb C0008. (**B & D)** As an example, sensorgrams (FC2-FC1) for C0008, ON103, and ON203 with human (**B**) and mouse (**D**) oxMIF (42-666 nM) including Langmuir 1:1 fitting curves (black lines) provided by the BiaEvaluation 4.1 software (Cytiva, RRID:SCR_015936) are shown.

Supplementary Figure S3

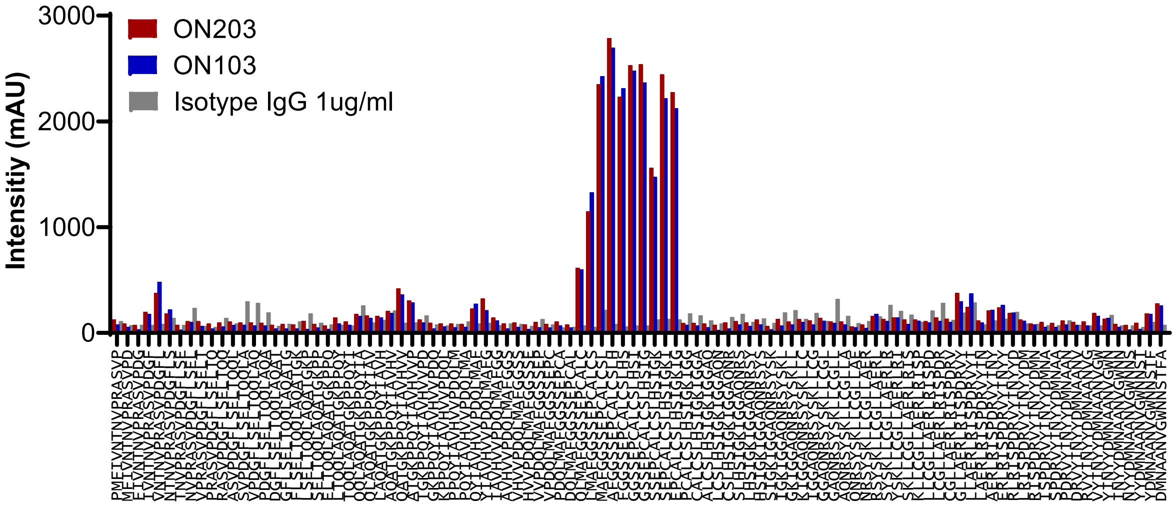

Supplementary Figure S3. Epitope mapping of ON103 and ON203 (25 ng/mL) on 1 amino acid overlapping 15mer linear peptides of human MIF. The full linear sequence of human macrophage migration inhibitory factor (MIF, UniProt ID P14174) is converted into a library of 15mer peptides each overlapping by 1 amino acid and directly synthesized on an amino-functionalized solid support as previously described in Timmerman et al. (6). Data are presented as mAU per 15mer peptide. An isotype IgG (1 µg/mL) was used as a negative control.

Supplementary Figure S4

A

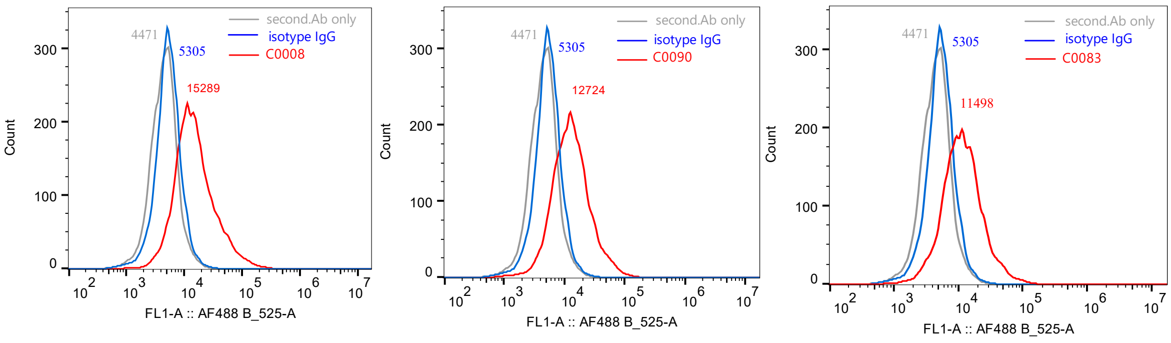

B

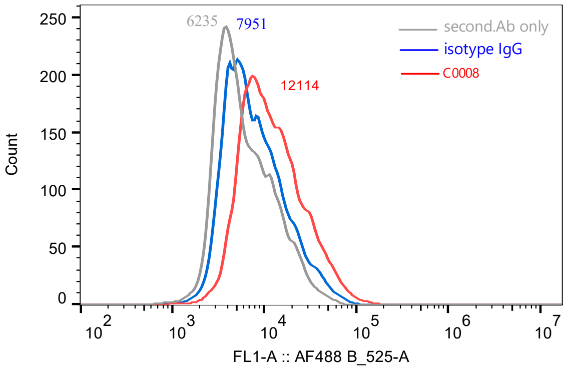

Supplementary Figure S4. Expression of oxMIF at the cell surface of PC-3 and HCT116 cancer cell lines. PC3 human prostate adenocarcinoma cells were stained with anti-oxMIF mAbs C0008, C0090, C0083, or isotype IgG at 75 nM (**A**); HCT116 human colon carcinoma cells were stained with anti-oxMIF mAb C0008, or isotype IgG at 75 nM (**B**). Bound mAbs were detected with polyclonal goat anti-human IgG (H+L) Alexa Fluor 488-conjugated antibody on CytoFlex-S flow cytometer, and data analyzed with FlowJo software. “Second. Ab only” histograms refer to the cells stained with goat anti-human IgG (H+L) Alexa Fluor 488-conjugated antibody only. Numbers on histogram overlays are geometric mean fluorescence intensity (MFI) values for AF488.

Supplementary Figure S5

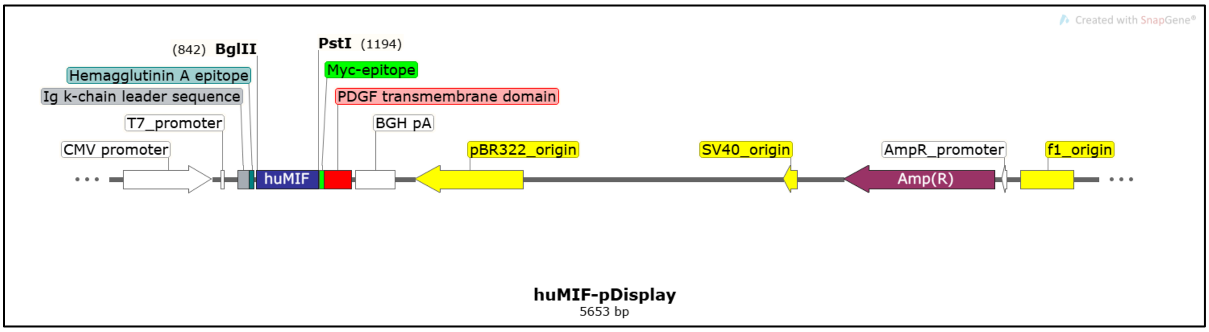

Supplementary Figure S5. Schematic presentation of the plasmid map (huMIF in pDisplay™ vector). The cDNA encoding human MIF was cloned into the pDisplay™ mammalian expression vector (Invitrogen/Thermo Fisher Scientific, catalog # V66020) under the pCMV promoter in-frame with N-terminal Hemagglutinin A epitope and C-terminal Myc-epitope by using B*gl* II (5′-) and *Pst* I (3′-) cloning sites. Due to the C-terminal transmembrane anchoring domain of platelet-derived growth factor receptor (PDGFR), after the removal of the signal peptide (IgG k-chain leader sequence), the monomeric huMIF-Myc-PDGFR fusion protein expressed from this construct is designed to be exposed at the cell surface of the transfected mammalian cells.

Supplementary Figure S6

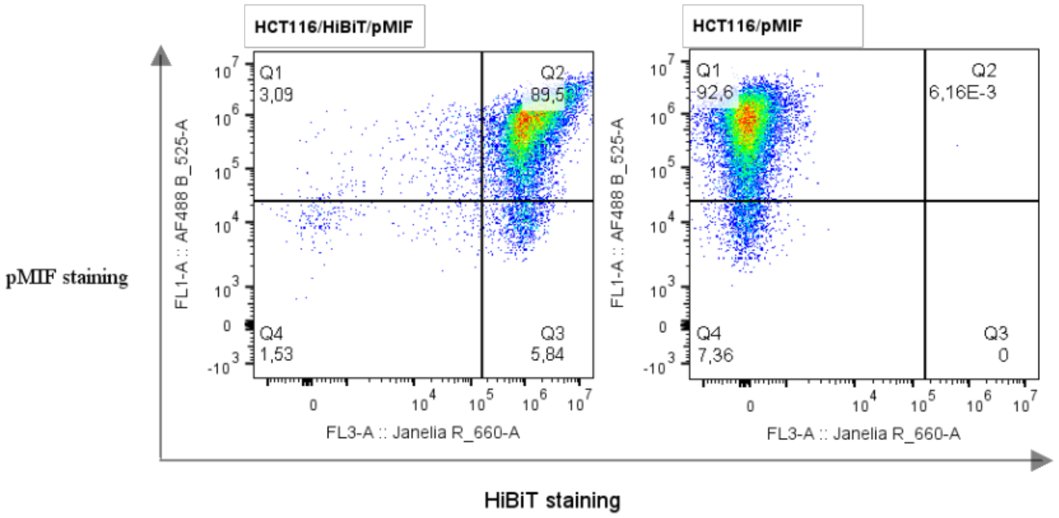

Supplementary Figure S6. Expression of huMIF on the cell surface of HCT116/pMIF and HCT116/HiBiT/pMIF stable cell lines. HCT116/pMIF and HCT116/HiBiT/pMIF cells were stained with Janelia Fluor 646-conjugated HaloTag® Ligand

(Promega) according to the manufacturer's instructions to allow the visualization of intracellular HiBiT. Subsequently, cells were stained with a polyclonal rabbit anti-human MIF antibody and a goat anti-rabbit Alexa Fluor 488-conjugated antibody (Thermo Fisher Scientific) for the detection of human MIF. Cells were analyzed on a CytoFlex-S flow cytometer (Beckman Coulter). HuMIF is exposed at the cell surface of the cell line stably transfected with huMIF-pDisplay plasmid (HCT116/pMIF, right dot blot, upper left quadrant (Q1)), and co-expression of huMIF and HiBiT-tagged HaloTag® protein could be seen in cell line stably expressing HiBiT that was then stably transfected with huMIF-pDisplay plasmid (HCT116/HiBiT/pMIF, left dot plot, upper right quadrant (Q2)).

Supplementary References

1. Schinagl A, Mirkina I, Kerschbaumer RJ, Thiele M. WO2022/069712A1: Improved anti-oxMIF antibodies with reduced aggregation potential and reduced hydrophobicity. World Intellectual Property Organization Internal Bureau; 2022; filed 1 Oct, 2021.
2. World Health Organization. International nonproprietary names for pharmaceutical substances (INN): proposed INN: list 120. WHO Drug Information. World Health Organization; 2018.
3. Foster P, Byrd J. WO2015195498A1: Treatment for chronic lymphocytic leukemia (CLL). World Intellectual Property Organization Internal Bureau; 2015. page 1–30.
4. Kabat EA. Sequences of proteins of immunological interest. 5th ed. US Department of Health and Human Services, Public Health Service, National Institutes of Health; 1991.
5. Edelman GM, Cunningham BA, Gall WE, Gottlieb PD, Rutishauser U, Waxdal MJ. The covalent structure of an entire γG immunoglobulin molecule. Proceedings of the National Academy of Sciences. National Acad Sciences; 1969;63:78–85.
6. Timmerman P., Puijk WC. and Meloenet RH. Functional reconstruction and synthetic mimicry of a conformational epitope using CLIPS™ technology. J. Mol. Recognit.; 2007;20:283–299.
